# Activation of endogenous glucocorticoids by HSD11B1 inhibits the antitumor immune response in renal cancer

**DOI:** 10.1080/2162402X.2023.2286820

**Published:** 2023-11-30

**Authors:** Hélène Poinot, Eloïse Dupuychaffray, Grégoire Arnoux, Montserrat Alvarez, Jérémie Tachet, Ounss Ezzar, Jonathan Moore, Olivia Bejuy, Eulalia Olesti, Gioele Visconti, Víctor González-Ruiz, Serge Rudaz, Jean-Christophe Tille, Clarissa D. Voegel, Patrycja Nowak-Sliwinska, Carole Bourquin, Aurélien Pommier

**Affiliations:** a School of Pharmaceutical Sciences, University of Geneva, Geneva, Switzerland; b Institute of Pharmaceutical Sciences of Western Switzerland, University of Geneva, Geneva, Switzerland; c Department of Cell Physiology and Metabolism, Faculty of Medicine, University of Geneva, Geneva, Switzerland; d Translational Research Centre in Oncohaematology, Faculty of Medicine, University of Geneva, Geneva, Switzerland; e CIBM Center for Biomedical Imaging, Faculty of Medicine, University of Geneva, Geneva, Switzerland; f Division of Clinical Pathology, Geneva University Hospitals, Geneva, Switzerland; g Department of Nephrology and Hypertension, Inselspital, Bern University Hospital, University of Bern, Bern, Switzerland; h Department of Anesthetics, Pharmacology, Intensive Care and Emergencies, Faculty of Medicine, University of Geneva, Geneva, Switzerland

**Keywords:** Glucocorticoids, HSD11B1, immunotherapy, renal cancer, steroidogenesis

## Abstract

Although immune-based therapies have revolutionized the management of cancer, novel approaches are urgently needed to improve their outcome. We investigated the role of endogenous steroids in the resistance to cancer immunotherapy, as these have strong immunomodulatory functions. Using a publicly available database, we found that the intratumoral expression of 11 beta-hydroxysteroid dehydrogenase type 1 (*HSD11B1*), which regenerates inactive glucocorticoids into active glucocorticoids, was associated with poor clinical outcome and correlated with immunosuppressive gene signatures in patients with renal cell carcinoma (RCC). HSD11B1 was mainly expressed in tumor-infiltrating immune myeloid cells as seen by immunohistochemistry in RCC patient samples. Using peripheral blood mononuclear cells from healthy donors or immune cells isolated from the tumor of RCC patients, we showed that the pharmacological inhibition of HSD11B1 improved the response to the immune checkpoint inhibitor anti-PD-1. In a subcutaneous mouse model of renal cancer, the combination of an HSD11B1 inhibitor with anti-PD-1 treatment increased the proportion of tumor-infiltrating dendritic cells. In an intrarenal mouse tumor model, HSD11B1 inhibition increased the survival of mice treated with anti-PD-1. In addition, inhibition of HSD11B1 sensitized renal tumors in mice to immunotherapy with resiquimod, a Toll-like receptor 7 agonist. Mechanistically, we demonstrated that HSD11B1 inhibition combined with resiquimod increased T cell-mediated cytotoxicity to tumor cells by stimulating the antigen-presenting capacity of dendritic cells. In conclusion, these results support the use of HSD11B1 inhibitors to improve the outcome of immunotherapy in renal cancer and highlight the role of the endogenous glucocorticoid metabolism in the efficacy of immunotherapy.

## Introduction

Cancer immunotherapy has revolutionized the field of oncology by prolonging patient survival. In clear cell renal cell carcinoma (ccRCC), immune checkpoint inhibitors are approved for first-line treatment of metastatic disease and have improved overall survival across multiple clinical trials.^1^ However, only 10% of patients with advanced ccRCC achieve complete response to checkpoint inhibitors.^2^ Since expression of PD-L1 does not predict response to treatment in renal cancer^3^, it is urgent to identify the factors of resistance to checkpoint inhibition and to develop novel combination therapies to improve the outcome of immunotherapy.

Steroid hormones such as glucocorticoids play a role in regulating various physiological functions, including the immune response.^4^ Following treatment with immune checkpoint inhibitors, exogenous glucocorticoids are prescribed to control immune-related adverse events, but whether endogenous steroid hormones influence the response to immunotherapy in ccRCC is unknown.^5^

Although systemic levels of steroids are mostly governed by their production in the gonads and the adrenal gland, their biological activity is highly regulated in peripheral tissues in which the final steps of steroidogenesis can occur. This regulation has been demonstrated in several tissues and cell types, including immune cells.^6^ In particular, glucocorticoids in peripheral tissues are activated by 11 beta-hydroxysteroid dehydrogenase type 1 (HSD11B1) and inactivated by 11 beta-hydroxysteroid dehydrogenase type 2 (HSD11B2), a mechanism which contributes to the local regulation of the stimulation of the glucocorticoid receptor.^7^ In cancer, evidence of intratumoral steroidogenesis was first shown in hormone-sensitive cancers such as breast and prostate tumors.^8,9^ More recently, the expression of genes involved in steroidogenesis was characterized as a signature for survival of patients with hormone-independent cancers such as gastric cancer and gastrointestinal stromal tumors.^10,11^

Since steroids are important modulators of the immune response, we investigated whether the enzymes involved in steroid metabolism were associated with clinical outcome and antitumor immune responses in ccRCC. Our results shed light on the inhibitory function of glucocorticoid regeneration through HSD11B1 in the immune response against renal cancer.

## Materials and methods

See Supplemental Information

## Results

### HSD11B1 *expression in tumors correlates with poor clinical outcome in patients with renal cancer*

**Figure 1.**
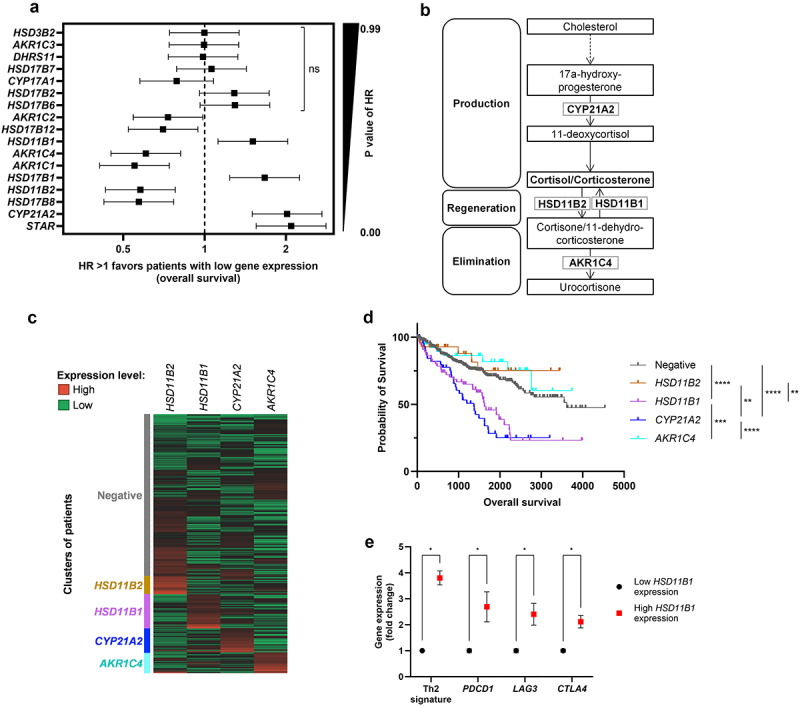
(a) forest plot showing the hazard ratio (HR) for overall survival (OS) in ccRCC patients with high versus low expression of genes involved in steroidogenesis. Patients were dichotomized into high vs. low gene expression groups based on the median expression values as threshold. HR is represented on a logarithmic scale, HR > 1 indicates that high expression of the gene correlates with a shorter OS. (b) simplified diagram of the glucocorticoid pathway. (C) heat map representing hierarchical clustering analysis of genes involved in glucocorticoid metabolism. (d) probability of survival for patients segregated based on the hierarchical clustering. **p-value <10^−2^, ***p-value <10^−3^, ****p-value <10^−4^. (e) expression levels for immune checkpoints and Th2 gene signature in patients with high vs. low HSD11B1 expression. Data are shown as mean ± SEM of 445 (low *hsd11b1* expression) and 57 patients (high *hsd11b1* expression), ****p-value <10^−6^.

In order to identify clinically impactful genes that influence the antitumor immune response, we correlated the intratumoral expression of genes involved in steroidogenesis with the outcome of ccRCC patients and looked for an association with immunosuppressive markers using the TCGA repository. We found that high expression of *HSD11B1*, *HSD17B1*, *CYP21A2* and *STAR* was associated with poor overall survival while on the contrary, high expression of *AKR1C2*, *HSD17B2*, *AKR1C4*, *AKR1C1*, *HSD11B2*, *HSD17B8* was associated with a longer overall survival (OS) in patients (Figure 1a). This analysis drew our attention to the glucocorticoid pathway, as four genes were related to cortisol metabolism (Figure 1b and S1a). The CYP21A2 and HSD11B1 enzymes support the production of cortisol, while HSD11B2 and AKR1C4 are involved in cortisol inactivation and degradation. This finding indicated that patients with high expression of cortisol-producing enzymes or a low expression of cortisol-inactivating enzymes had reduced OS. Hierarchical clustering analysis revealed five main patient populations with one cluster of patients expressing low levels of these enzymes and four clusters mainly defined by the strong expression of each individual gene, demonstrating that *HSD11B1*, *CYP21A2*, *AKR1C4* and *HSD11B2* were mutually exclusively expressed in ccRCC patients (Figure 1c). Survival analyses of the patient populations defined by the hierarchical clustering clearly showed that patients defined by high expression of *HSD11B1* or *CYP21A2* had a negative outcome compared to the patients defined by high expression of *AKR1C4* or *HSD11B2* (Figure 1d). Since *CYP21A2* is not only involved in the cortisol synthesis pathway but also regulates the mineralocorticoid synthesis pathway, we then focused our analysis on *HSD11B1*, which is directly and specifically involved in cortisol regeneration. The log-rank test showed a greater impact of HSD11B1 on survival when the patient population was defined by the clustering analyses (HR = 2.187, 95%CI: 1.511–3.166) (Figure S1b) compared to the median-based method (HR = 1.3, 95%CI:1.17–1.45) (Figure 1a). Among the 39 cancer subtypes tested in TCGA, the expression of HSD11B1 was found to correlate with a poor prognosis only in stomach adenocarcinoma and renal cancer. Renal cancer was the cancer type most significantly associated with shorter OS (Figure S2).

Since HSD11B1 is involved in cortisol regeneration, which may lead to local immunosuppression,^12^ we hypothesized that ccRCC patients defined by a high *HSD11B1* could be associated with an immunosuppressive gene expression pattern. Interestingly, patients with high expression of *HSD11B1* had a higher expression of *PDCD-1* (coding for PD-1), *LAG-3*, *CTLA-4* and genes involved in a Th2 immune response (Figure 1e).

**Figure 2.**
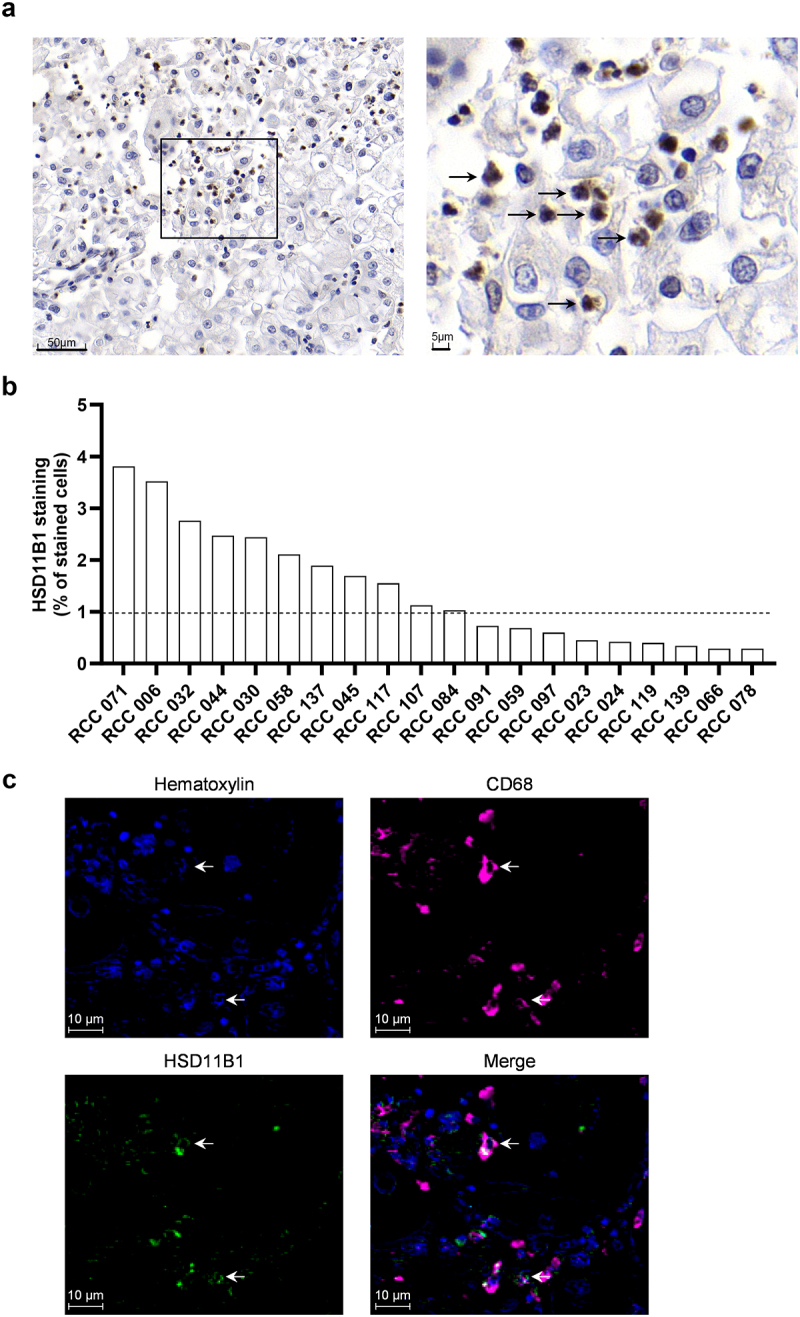
(a) human RCC sample from tumor block stained for HSD11B1 (brown) by immunohistochemistry. The right picture is a zoom in section. The arrows indicate infiltrating cells positive for HSD11B1 with a macrophage morphology. (b) percentage of cells positive for HSD11B1 in tumors from 20 patients with RCC. (c) representative immunohistochemistry staining for CD68, HSD11B1 and hematoxylin in four RCC tumor specimens from patients. Arrows show colocalization of HSD11B1 and CD68.

In order to confirm the expression of HSD11B1 at the protein level in renal tumors and to identify the positive cells, we stained twenty tumors from ccRCC patients by immuno-histochemistry. As shown in Figure 2a, a positive staining of HSD11B1 was mainly detected in infiltrating cells with 11/20 patients showing more than 1% of HSD11B1-positive cells, that were mainly found in the core of the tumor (Figure 2b and Figure S3). HSD11B1 staining was not correlated with survival in this small 20-patient cohort. Morphological analysis of the staining by a pathologist suggested that HSD11B1-positive cells were immune cells of the myeloid lineage, especially macrophages and neutrophils (Figure S3). Co-staining of HSD11B1 with CD68^+^ cells confirmed that HSD11B1 was expressed in tumor-infiltrating macrophages (Figure 2c).

### HSD11B1 activity inhibits antigen-mediated T cell activation and limits the response to anti-PD-1 treatment in human PBMC and tumor-infiltrating immune cells

**Figure 3.**
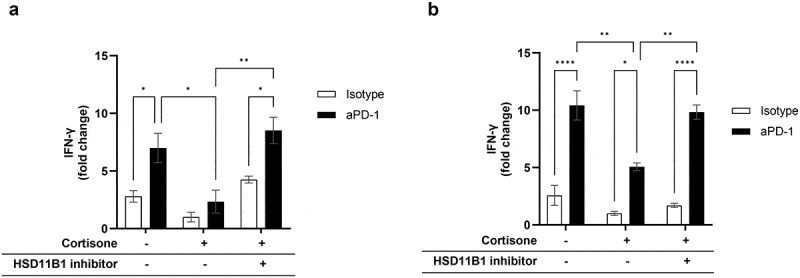
Relative IFN-γ levels secreted by PBMC from a healthy donor (a) and immune cells enriched from a human RCC tumor (b), following antigen recall stimulation. In absence of cortisone, there was no effect of ABT-384 on the level of IFN-γ in stimulated or non-stimulated conditions (data not shown). Fold change compared to the condition with cortisone, isotype and without HSD11B1 inhibitor is represented as mean ± SEM of 3 technical replicates in one experiment. *p-value <0.05, **p-value <10^−2^, ***p-value <10^−3^, ****p-value <10^−4^. A. Data are representative of 3 independent experiments on the same donor. Similar results were obtained with 2 other donors tested in one experiment. B. out of 4 patients tested, only the patient shown had a response to antigen recall.

A key function of myeloid cells is to stimulate the adaptive T cell response against cancer.^13^ We tested the functional impact of HSD11B1 activity in peripheral blood mononuclear cells (PBMCs) healthy human donors and human immune cells isolated from RCC tumors. An antigen recall assay was performed to test whether HSD11B1 could impact antigen-dependent T cell activation and the response to anti-PD-1 treatment in these cells. T cell activation, measured by IFN-γ secretion, was detected only in the presence of antigen stimulation and was increased by anti-PD-1 treatment. Treatment with cortisone, an inactive HSD11B1 substrate, reduced T cell activation both in isotype and anti-PD-1 treated conditions, suggesting that immune cells were able to metabolize cortisone into the active hormone cortisol through HSD11B1. The inhibitory effect of cortisone on T cell activation was reversed using ABT-384, an HSD11B1 inhibitor, demonstrating that the cortisone-mediated inhibition of the immune response was driven by HSD11B1 (Figure 3a). Interestingly, while treatment with cortisone blocked the efficacy of anti-PD-1 treatment in this assay, the addition of ABT-384 restored T cell activation toward a level comparable with the non-cortisone-treated condition.

The effect of HSD11B1 activity was then tested on immune cell subsets isolated from RCC tumors. Although only one out of four patients tested showed a response to antigen recall, we observed a reduction of the T cell response in cortisone-treated samples in both isotype and anti-PD-1 conditions, which was reversed by ABT-384 (Figure 3b). These results demonstrated that HSD11B1 can inhibit the antigen-specific response in tumor-infiltrating immune cells.

### HSD11B1 inhibition impacts the immune phenotype in anti-PD-1-treated subcutaneous renal tumors in mice

**Figure 4.**
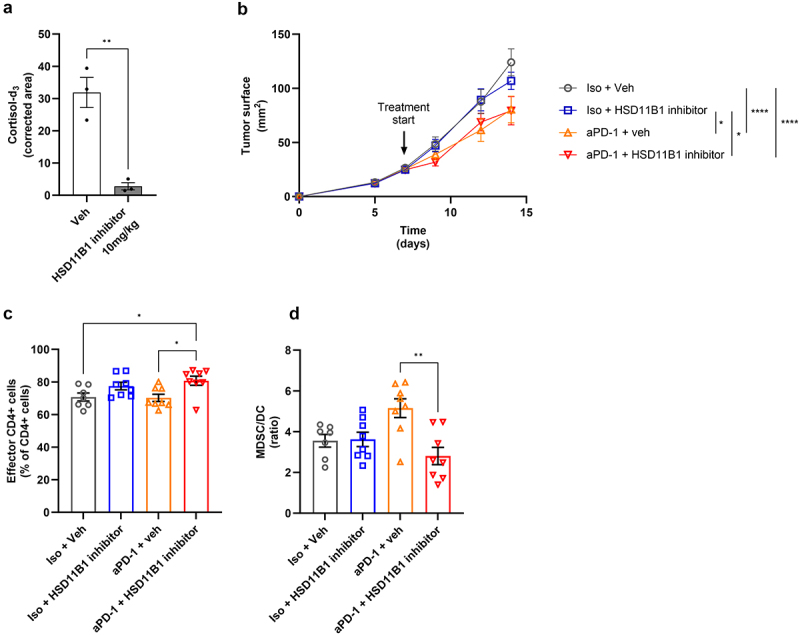
(a) determination of HSD11B1 activity by the measurement of plasma cortisol-d_3_ levels in naive mouse after administration of cortisol-d4 ± the HSD11B1 inhibitor ABT-384. Data are shown as mean ± SEM of 3 mice per group. **p-value <10^−2^. (b) growth kinetics of subcutaneous Renca tumors. Data are shown as mean of tumor area ± SEM of 8 mice per group. Statistical analysis at day 14, *p-value <0.05, ****p-value <10^−4^. (c-d) immunophenotyping results of the tumor by FACS. CD44^−^CD62L^−^ CD4^+^ were defined as effector CD4^+^ cells and are represented as % of total CD4^+^ cells. (c) MDSC/DC ratio calculated based on the % of MDSC/CD45^+^ cells and % of DC/CD45^+^ cells (figureS6). (d) Data are shown as mean ± SEM of 8 mice per group. *p-value <0.05, **p-value <10^−2^. Animal experiments were performed once.

Since HSD11B1 inhibition improved the T cell response to an immune checkpoint inhibitor *in vitro*, we tested the therapeutic potential of combining an HSD11B1 inhibitor with anti-PD-1 in mice. First, cortisol-d4 was administered to non-tumor-bearing mice treated with ABT-384 and HSD11B1 activity was quantified. We observed a complete inhibition of HSD11B1 activity as demonstrated by the low level of cortisol-d_3_ measured in the plasma of inhibitor-treated mice (Figure 4a). The Renca cell line, which expressed the highest levels of *Hsd11b1* and *Hsd11b2* compared to mouse melanoma, breast cancer, colon cancer and hepatoma cell lines (Figure S5), was selected as a syngeneic renal cancer model for in vivo studies. CYP21A2 and AKR1C4 were not detected in this model (data not shown). In mice bearing subcutaneous murine renal cancer (Renca) tumors, anti-PD-1 treatment partially inhibited tumor growth. No efficacy of monotherapy with the HSD11B1 inhibitor was observed when compared to the vehicle-treated control group (Figure 4b). Furthermore, there was no effect of the combination treatment with the HSD11B1 inhibitor and anti-PD-1 on tumor growth compared to the anti-PD-1 treated group. Regarding the tumor immune phenotype, intratumoral CD4^+^ effector cells were increased in the combination group compared to the anti-PD-1 monotherapy group (Figure 4c). In addition, a decrease of the myeloid-derived suppressor cell to dendritic cell ratio (MDSC/DC) in the combination group compared to anti-PD-1 monotherapy-treated group was observed (Figure 4d and Figure S6). Thus, although HSD11B1 inhibition did not confer higher antitumoral efficacy to anti-PD-1 treatment, the combination impacted the tumor immune phenotype.

### Combination of an HSD11B1 inhibitor with anti-PD-1 therapy increases the survival of mice bearing orthotopic renal tumors

**Figure 5.**
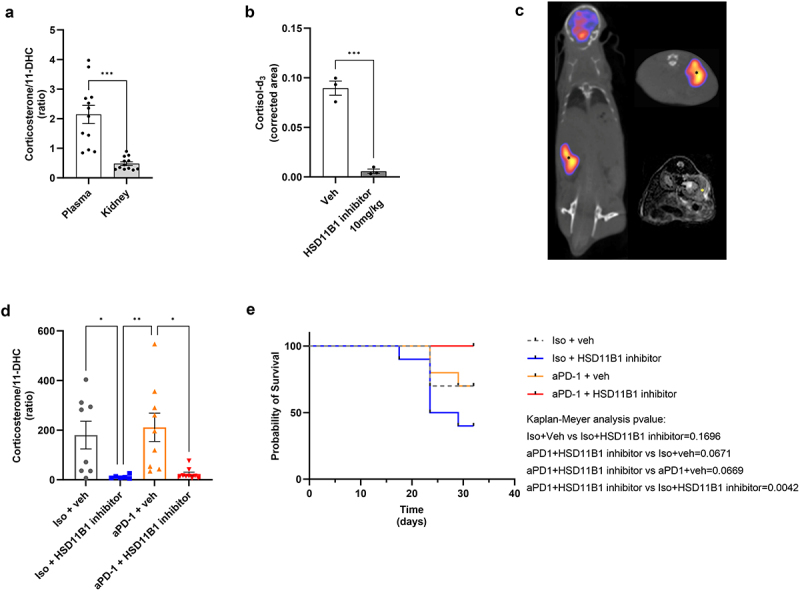
(a) comparison of corticosterone/11-DHC ratio in plasma and kidney of naive mice. Data are shown as mean ± SEM of 12 mice per group. ***p-value = 5×10^−4^. (b) determination of HSD11B1 activity by the measurement of cortisol-d_3_ levels in kidneys of naive mice after administration of cortisol-d4 ± the HSD11B1 inhibitor ABT-384. Data are shown as mean ± SEM of 3 mice per group. ***p-value <10^−3^. (c) representative images of a mouse with an intrarenal Renca tumor. Coronal (left) and axial views (upper right) of the^15^F]FDG PET/CT scan (back of the mouse on the top). The lower right image corresponds to the axial view of the MRI scan. Tumor is pointed by the asterisks. (d) Corticosterone/11-DHC ratio in plasma of renal tumor-bearing mice. Data are shown as mean ± SEM of 6 to 9 mice per group. *p-value <0.05, **p-value <10^−2^. (e) probability of survival over time in mice bearing intrarenal Renca tumors based on the tumor volume. Tumor volume >200 mm^3^ considered as event of death, 10 mice per group. Statistical analysis: log-rank (Mantel-Cox) test. Animal experiments were performed once.

As HSD11B2 provides the substrates for HSD11B1 and higher levels of HSD11B2 activity have been described in the kidney compared to other tissues,^14^ we hypothesized that the inhibition of HSD11B1 could be more impactful in an orthotopic model of renal cancer. We observed a lower ratio of the murine active hormone corticosterone to the inactive murine precursor 11-dehydrocorticosterone (11-DHC) in the kidney than in the plasma of naive mice, confirming a predominant activity of HSD11B2 in kidneys (Figure 5a). We then confirmed that administration of ABT-384 inhibited HSD11B1 activity in the kidney of naive mice (Figure 5b). The combination of the HSD11B1 inhibitor with anti-PD-1 was then tested in mice bearing orthotopic Renca tumors and tumor growth was assessed by MRI and PET scans (Figure 5c). Treatment with ABT-384 decreased the corticosterone-to-11-DHC ratio in the plasma of tumor-bearing mice (Figure 5d). The survival of the mice treated with anti-PD-1 was not different from the isotype control group, indicating that anti-PD-1 was not effective in this model (Figure 5e and S7a). The probability of survival at day 32 was 100% for mice treated with the combination treatment, 70% for the control group or the mice treated with anti-PD-1 monotherapy and 40% in mice having received the HSD11B1 inhibitor monotherapy. Thus, a decrease of corticosterone levels through HSD11B1 inhibition may improve the efficacy to anti-PD-1 treatment in this model, although the difference was not significant. Interestingly, a higher corticosterone-to-11-DHC ratio was associated with an increased tumor size in the combination group only, suggesting that low levels of corticosterone correlated with an improved response to anti-PD-1 treatment (Figure S7b–e). These results indicated that a decrease of corticosterone levels through HSD11B1 inhibition may improve the efficacy of anti-PD-1 treatment in mice bearing intrarenal tumors.

### HSD11B1 inhibition confers sensitivity to the innate immune stimulating agent resiquimod in mice bearing subcutaneous Renca tumors

**Figure 6.**
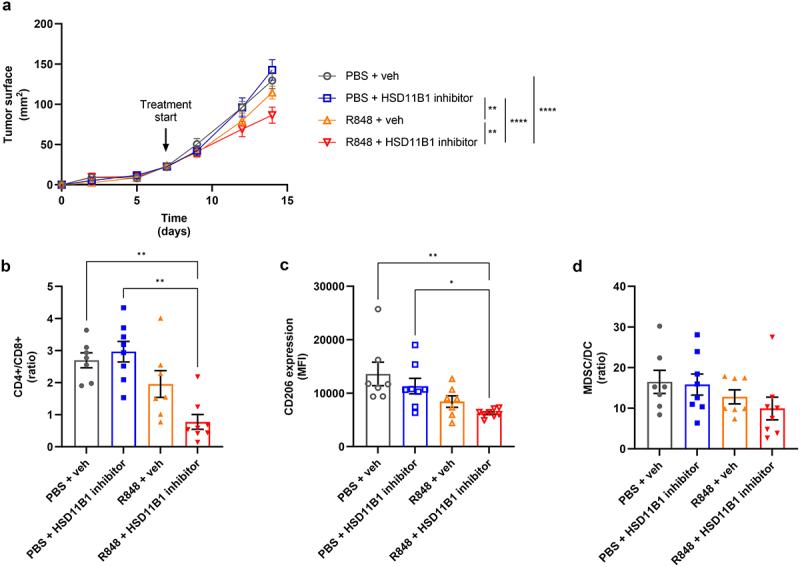
(a) growth kinetics of subcutaneously implanted Renca tumors. Treatments were initiated at day 7. Data are shown as mean of tumor area ± SEM of 7 to 8 mice per group. Statistical analysis at day 14, **p-value <10^−2^, ****p-value <10^−4^. (b-d) immunophenotyping results of the tumors by flow cytometry. Percentage of CD4^+^, CD8^+^ (b), MDSC, and DC (d) represented as ratio of cell populations. CD206 expression (c) represented as mean fluorescence intensity (MFI) of CD206 on macrophages in the tumor. Data are shown as mean ± SEM of 7 to 8 mice per group. *p-value <0.05, **p-value <10^−2^. Animal experiments were performed once.

As HSD11B1 was mainly expressed in myeloid cells of RCC patients, and since we observed an increase of the MDSC/DC ratio in response to HSD11B1 inhibition in anti-PD-1-treated tumors, we tested whether HSD11B1 activity modulated the anti-tumor response to resiquimod (R848), which is a TLR7/8 agonist. We have previously shown that R848 enhances antitumor CD8+ responses and decreases intratumoral myeloid-derived suppressor cells.^16,17^ Quantification of the corticosterone-to-11-DHC ratio showed no effect of R848 treatment on the plasma concentration of these steroids (data not shown). In tumor-bearing mice, although no reduction of tumor growth was observed either with R848 or with ABT-384 as monotherapies, the combination of both led to a decrease in tumor size at 14 days post-engraftment (Figure 6a). Interestingly, a reduction in tumor-infiltrating CD4^+^ T cells and an increase in tumor-infiltrating CD8^+^ T cells were observed in the combination group, as seen by the significant decrease of the CD4^+^/CD8^+^ ratio (Figure 6b and S8b,c). In addition, the combination treatment induced a decrease of the mannose receptor (CD206) protein expression in tumor-infiltrating macrophages, suggesting a switch toward a proinflammatory phenotype (Figure 6c). Although not significant, the combination therapy was also associated with a slight reduction in the MDSC/DC ratio (Figure 6d). These results suggested that stimulation of the myeloid compartment by a TLR7/8 agonist in combination with HSD11B1 inhibition improves the antitumor immune response.

### HSD11B1 inhibits T cell-mediated tumor cytotoxicity by down-regulating the activation of myeloid cells

**Figure 7.**
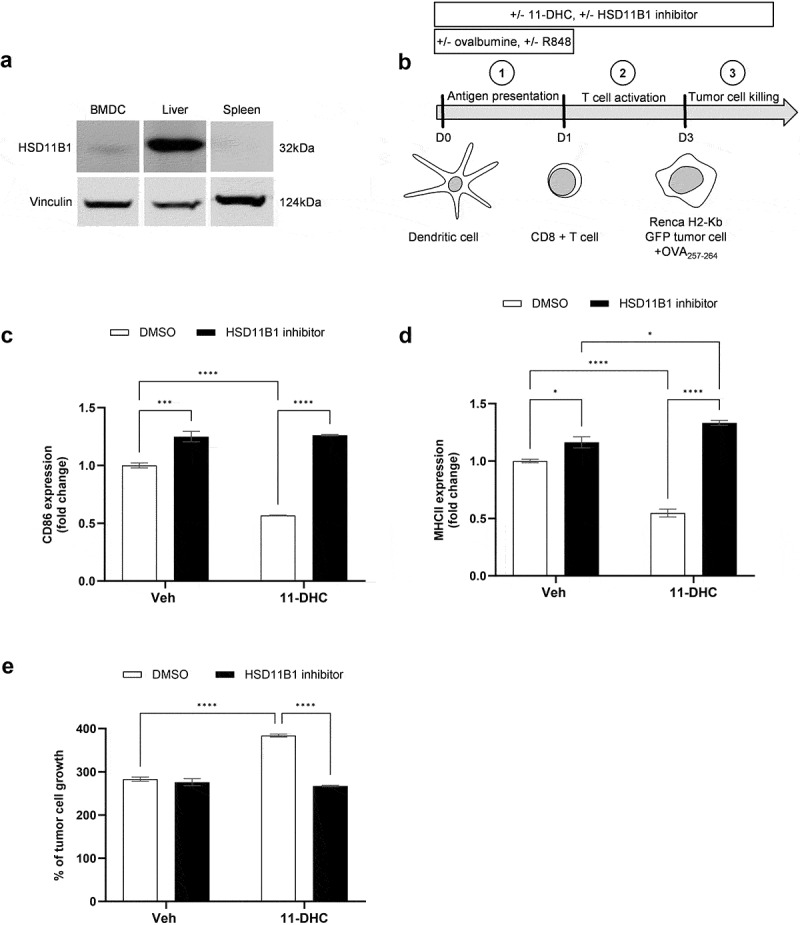
(a) HSD11B1 expression by Western blot in mouse bone marrow-derived dendritic cells, liver and spleen. (b) experimental design of T cell-mediated tumor cytotoxicity assay. C, D. surface CD86 (c) and MHCII (d molecules on bone marrow-derived dendritic cells measured by flow cytometry at D1. Results are expressed as fold change of the MFI, relative to the control condition. Data are shown as mean ± SEM of 3 technical replicates of one experiment. (e) measurement of Renca H2-kb GFP tumor cell growth exposed to antigen stimulated CD8^+^ T cells. Results are expressed as % of tumor cell growth normalized to the seeding density. Data are shown as mean ± SEM of 3 replicates and representative of 3 independent experiments. *p-value <0.05, ***p-value <10^−3^, ****p-value <10^−4^.

In order to better characterize the effect of HSD11B1 inhibition on the antitumor immune response in the context of TLR7 stimulation, we tested the impact of HSD11B1 activity on the capacity of mouse dendritic cells to initiate an antitumor immune response against renal cancer cells. HSD11B1 expression was previously described in human dendritic cells and in human and mouse macrophages,^15,18–20^. We confirmed HSD11B1 expression in mouse bone marrow-derived dendritic cells (BMDC) (Figure 7a). Dendritic cells were activated by R848 to prime cytotoxic T cells (Figure 7 B).^21^ Treatment with 11-DHC led to downregulation of surface CD86 and MHCII indicating decreased BMDC activation, which was reversed by HSD11B1 inhibition (Figure 7c,d). CD8^+^ T cells and Renca H2-kb GFP cells were then cocultured with the dendritic cells (Figure 7b). Exposure to 11-DHC enhanced tumor cell growth, suggesting that myeloid cells primed T-cell-mediated cytotoxicity against tumor cells less efficiently (Figure 7e). HSD11B1 inhibition restored the antitumor immune response in the presence of 11-DHC to the levels seen with the vehicle-treated control (Figure 7e). In the absence of immune cells, the growth of Renca H2-kb GFP cells did not change in response to 11-DHC or HSD11B1 inhibition (data not shown). These results demonstrated that BMDC are sensitive to 11-DHC and that HSD11B1 inhibits the anti-cancer immune response through a downregulation of the antigen presentation capacity of the dendritic cells.

## Discussion

Unprecedented advances have been made in the treatment of renal cancer through the use of immune checkpoint blockade, but some drivers of resistance are still unknown. In RCC a high CD8^+^ T cell infiltration is correlated with a worse prognosis and has not been associated with a higher probability of response to anti-PD-1 therapy, in contrast to what is seen in many other types of cancer.^22,23^ To the best of our knowledge, it is not fully understood why inflamed renal tumors do not respond well to immune checkpoint inhibitors, although a low tumor mutational burden and specific somatic mutations may play a role.^23,24^ Here we propose HSD11B1 as a novel factor contributing to resistance to immunotherapy in renal cancer.

Among all the cancer types available in the TCGA database, the poor prognostic value of intratumoral *HSD11B1* expression was seen mainly in patients with renal cancer. O

ne explanation may be the high level of activity in the kidney of its isoenzyme HSD11B2, which inactivates cortisol or corticosterone into cortisone or 11-DHC and thus produces the substrate for HSD11B1.^14,25^ Accordingly, we observed a lower corticosterone/11-DHC ratio in the kidney than in the plasma of mice. The therapeutic efficacy of HSD11B1 inhibition in combination with immunotherapy was higher for orthotopic Renca tumors than for subcutaneous tumors, supporting the hypothesis that HSD11B1 activity may have more impact in the kidney, where higher levels of its substrates are present than in other organs. Consistently with this concept, we found that *HSD11B1* expression was also associated with poor prognosis in stomach adenocarcinoma, and it has been shown that *HSD11B2* is also expressed in the gastrointestinal tract.^26^ In addition, *HSD11B1* overexpression or gain mutations were associated with poor clinical outcome in patients with gastrointestinal stromal tumors.^11^

The inhibition of HSD11B1 may have a different impact according to the type of immunotherapy, as we observed a greater benefit of HSD11B1 inhibition for combination treatment with R848 than with anti-PD-1 immunotherapy in subcutaneous models. This may be due to the mode of action of R848 which activates myeloid cells through TLR7, in contrast to anti-PD1 treatment which acts directly on T cell function. We propose that HSD11B1 contributes to cancer-associated immunosuppression by regulating the activation of myeloid cells and their capacity to induce an effective T cell response. This is supported by the expression pattern of HSD11B1, which we found mainly in tumor-infiltrating macrophages in human RCC samples, in agreement with previous observations in RCC and in melanoma.^27,28^ Consistently with our results, a recent study reported a higher infiltration of macrophages in RCC samples expressing a higher level of *HSD11B1*, supporting the link between HSD11B1 expression and myeloid cell in the tumor microenvironment.^27^ In mice, we observed a decrease in expression of the anti-inflammatory marker CD206 on tumor-infiltrating macrophages in response to HSD11B1 inhibition, suggesting a shift toward a more inflammatory phenotype. Indeed, we showed that HSD11B1 inhibition improved the ability of both human and mouse myeloid cells to prime T cells. In the context of vaccination, inhibition of HSD11B1 was shown to synergize with CpG, a TLR9 agonist, to enhance T cell responses, suggesting that the amplification of endogenous glucocorticoids by HSD11B1 is an important mechanism for the regulation of the activity of antigen-presenting cells.^29^

In tumors, activation of the glucocorticoid receptor by its ligand leads to a decrease in the expression of co-stimulatory molecules such as *CD28* and of pro-inflammatory cytokines, and to an increased expression of immune checkpoints such as *PDCD1* (PD-1), *CTLA-4*, and *TIM3*. ^30^ This is consistent with our observation that immune checkpoints are more highly expressed in RCC tumors with elevated *HSD11B1* expression. Within tumor-infiltrating CD8^+^ T cells, the glucocorticoid receptor was shown to be highly expressed in exhausted CD8^+^ T cells and to promote the upregulation of genes associated with T cell dysfunction.^31^ A recent study showed that HSD11B1-generated glucocorticoids by tumor cells promote resistance to immunotherapy through the stimulation of Treg function^32^. Thus, glucocorticoid receptor activity is associated with the suppression of CD8^+^ T cell responses.

The functional relevance of the immunosuppression induced by endogenous glucocorticoids is supported by the emerging link between psychological stress and the outcome of cancer immunotherapy. Production of systemic glucocorticoids limits antitumor immune responses by abrogating type I interferon responses in dendritic cells.^33^ This mechanism is in line with our results showing that suppression of glucocorticoid production in dendritic cells through HSD11B1 inhibition supports their ability to prime a cytotoxic T cell response. In addition, our findings are consistent with a recent study demonstrating that HSD11B1 limits the response to PD-1 blockade in melanoma.^28^

In conclusion, this work supports the hypothesis that the combination of an HSD11B1 inhibitor with immune checkpoint blockade may be beneficial in renal cancer patients, highlighting the role of endogenous glucocorticoid metabolism in the efficacy of immunotherapy. Although not used in the clinic to date, HSD11B1 inhibitors have been widely developed in the framework of metabolic diseases.^34^ Our study provides a rationale to further investigate whether these inhibitors could be repurposed for the treatment of cancer by boosting antitumor immunity.

## Supplementary Material

Figure S9B.tif

Figure S9C.tif

Figure S8.tif

Figure S2.tif

Figure S4_2nd_rev.tif

Tables of supplementary material and method.docx

Figure S5.tif

Figure S1.tif

Poinot et al Supplemental_text_2nd_rev.docx

Figure S7.tif

Figure S9A.tif

Figure S3.docx

Figure S6.tif

## Data Availability

The data that support the findings of this study are available from the corresponding authors [CB, AP], upon reasonable request.
