# Supplementary figures and images for: Activation of endogenous glucocorticoids by HSD11B1 inhibits the antitumor immune response in renal cancer
Source: Oncoimmunology. 2023 Nov 30;13(1):2286820. doi: 10.1080/2162402X.2023.2286820 (PMC10761155; doi:10.1080/2162402X.2023.2286820)

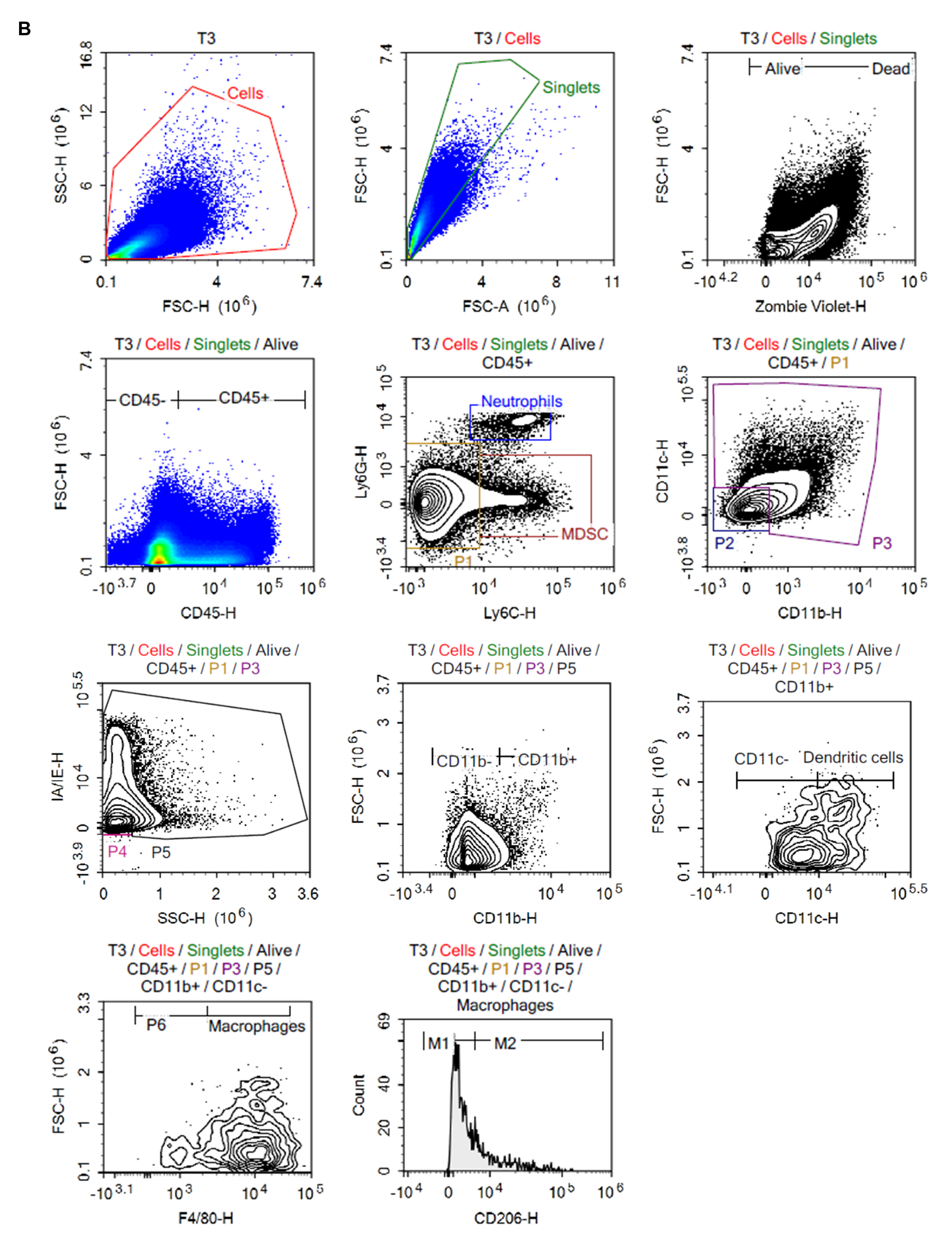

Supplement: Figure S9B.tif [file KONI_A_2286820_SM8820.tif]

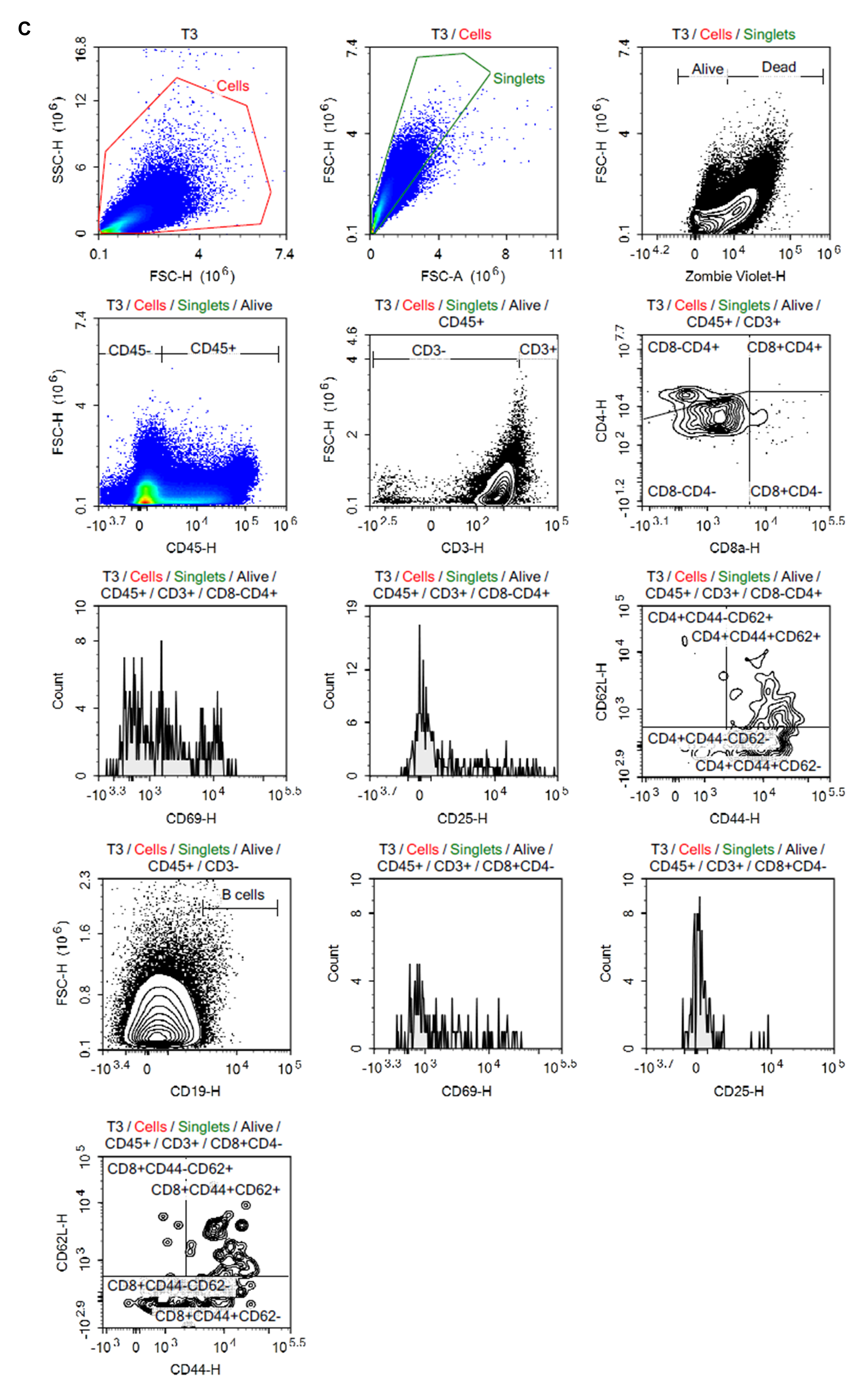

Supplement: Figure S9C.tif [file KONI_A_2286820_SM8819.tif]

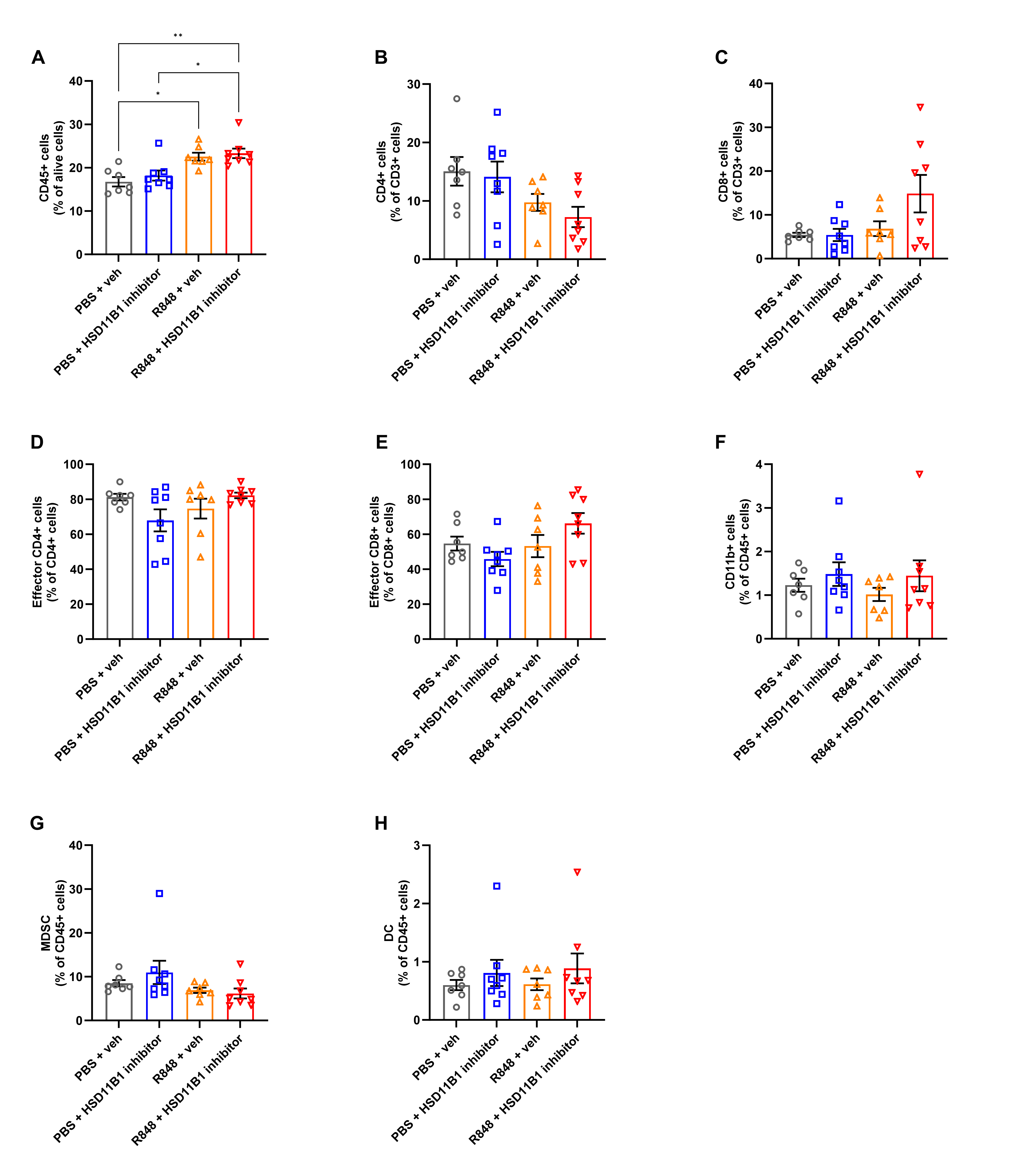

Supplement: Figure S8.tif [file KONI_A_2286820_SM8818.tif]

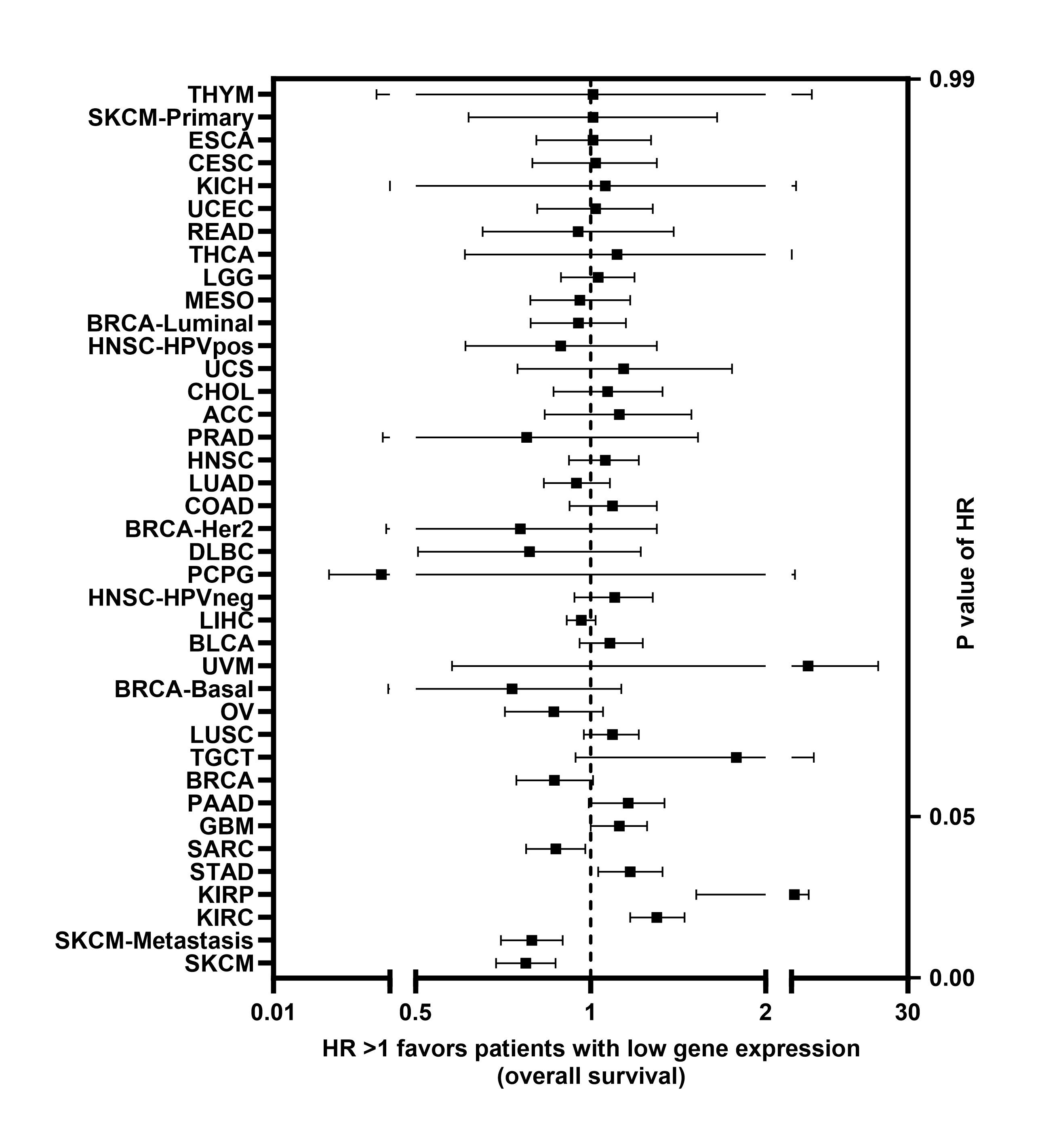

Supplement: Figure S2.tif [file KONI_A_2286820_SM8817.tif]

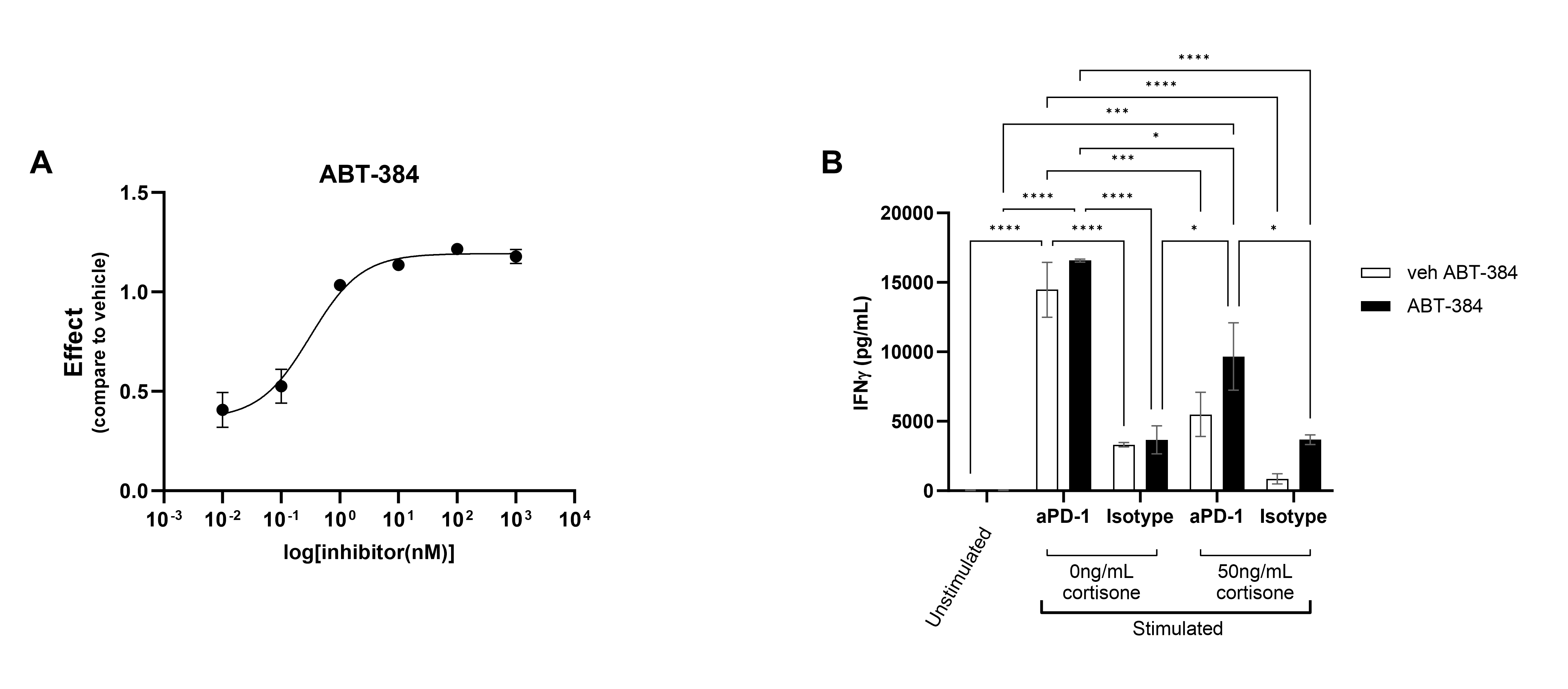

Supplement: Figure S4_2nd_rev.tif [file KONI_A_2286820_SM8816.tif]

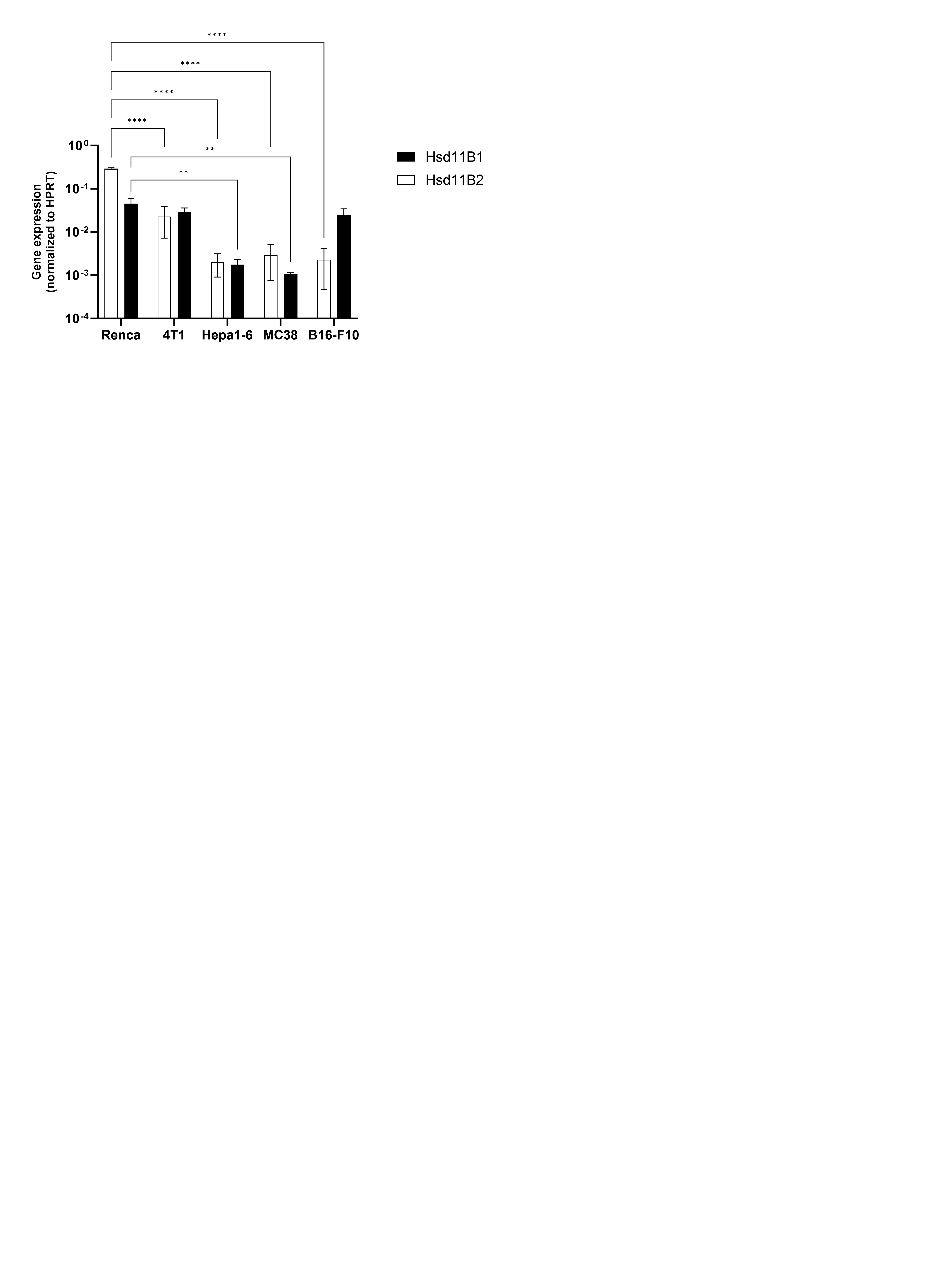

Supplement: Figure S5.tif [file KONI_A_2286820_SM8814.tif]

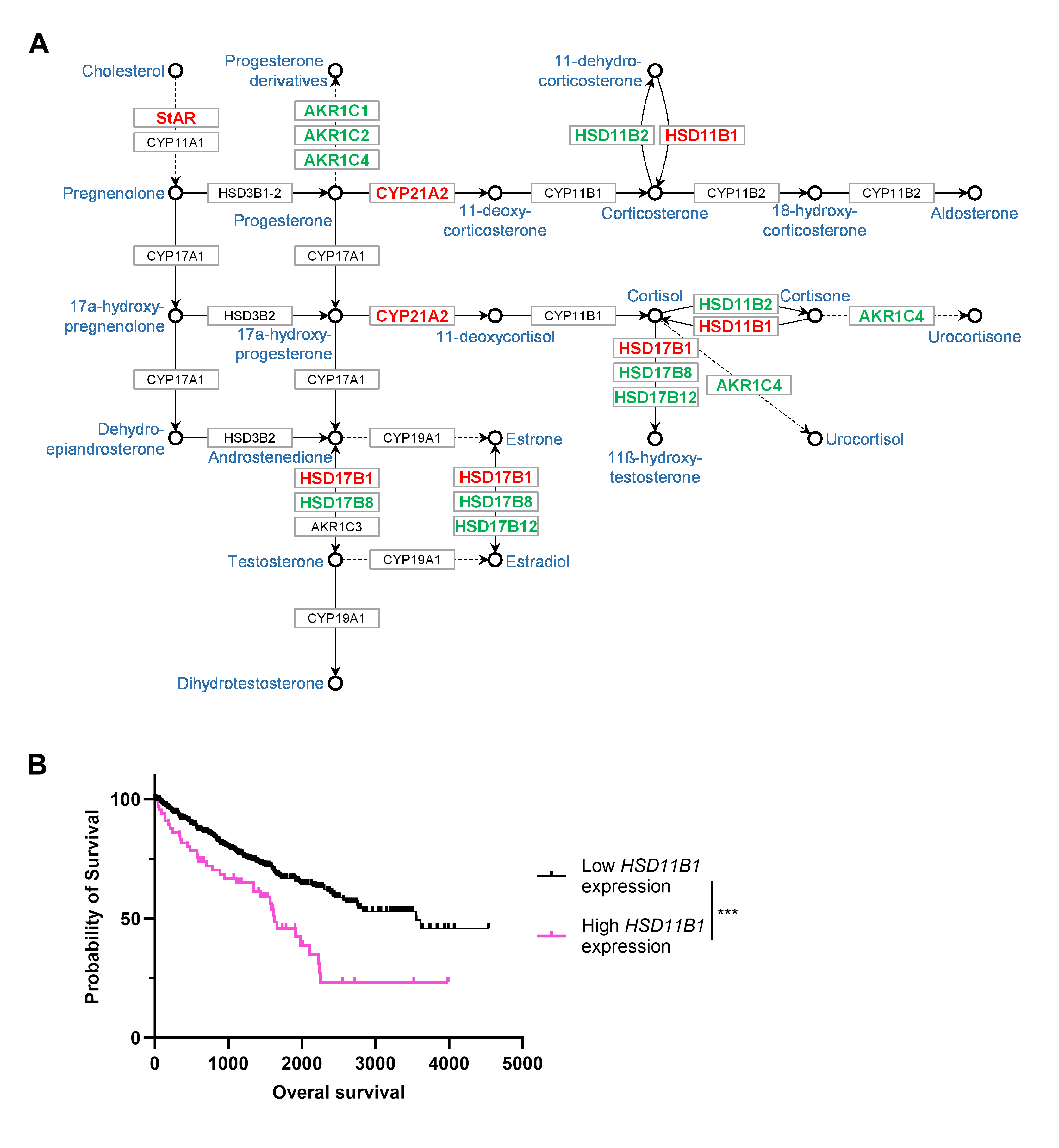

Supplement: Figure S1.tif [file KONI_A_2286820_SM8813.tif]

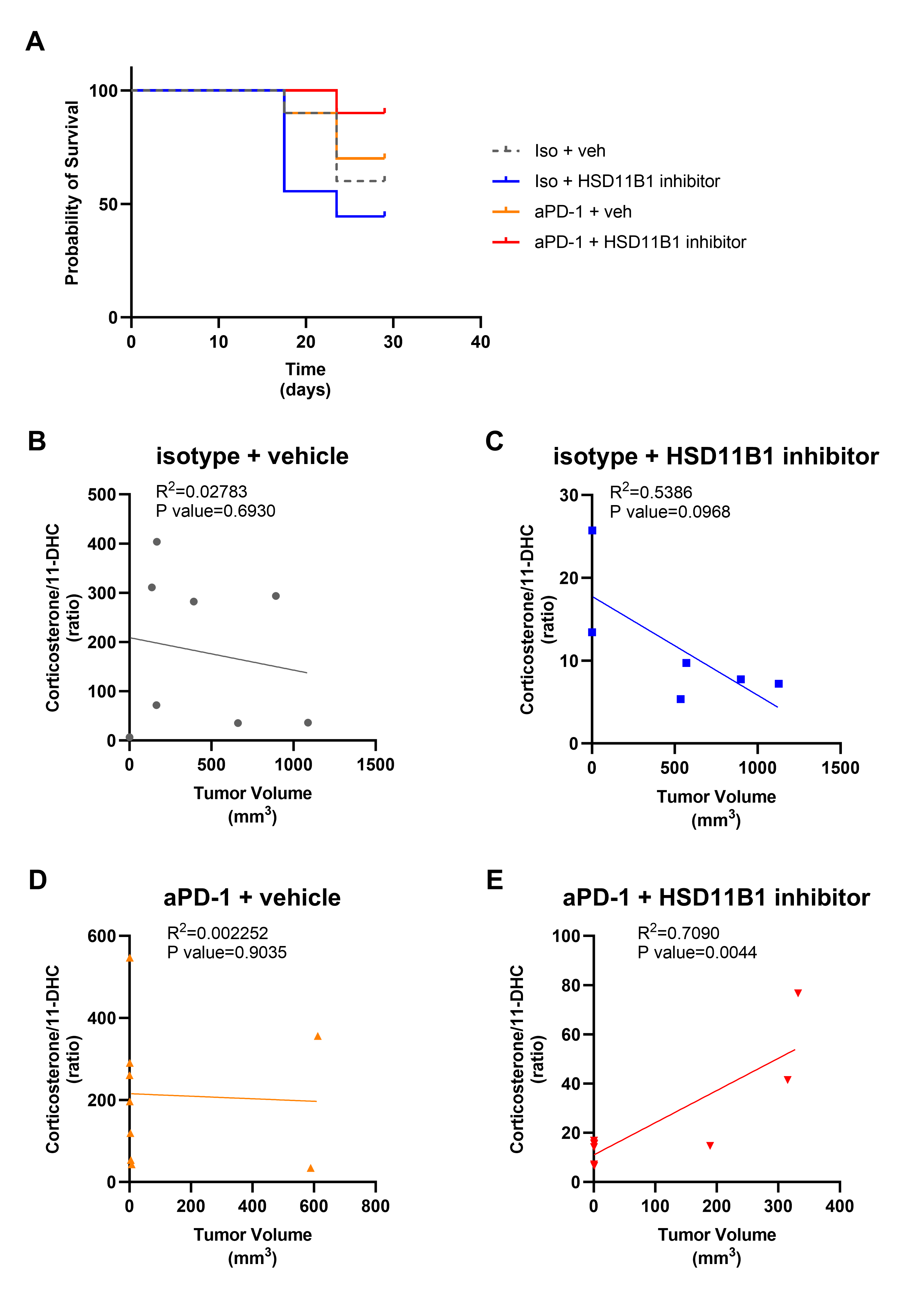

Supplement: Figure S7.tif [file KONI_A_2286820_SM8811.tif]

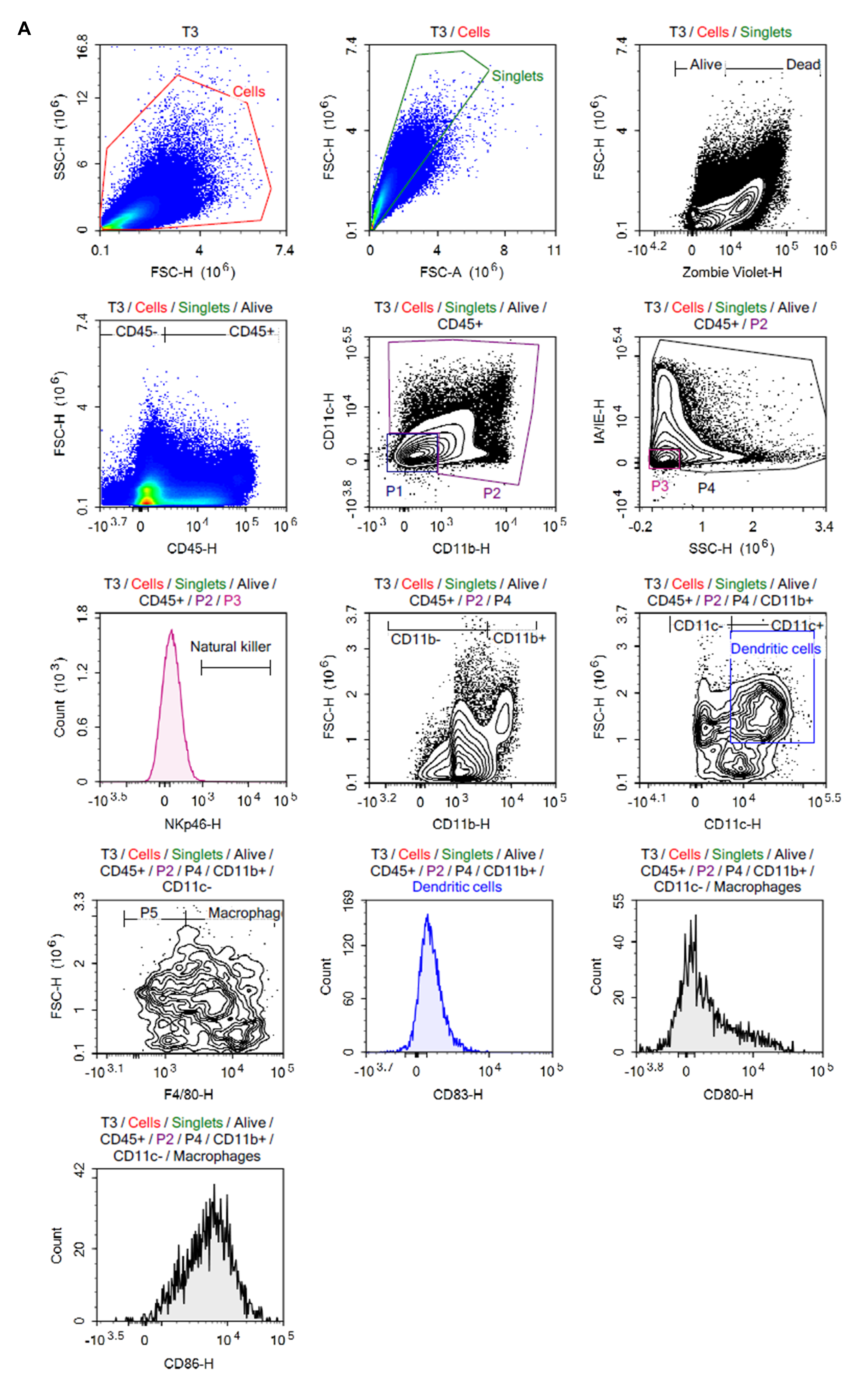

Supplement: Figure S9A.tif [file KONI_A_2286820_SM8810.tif]

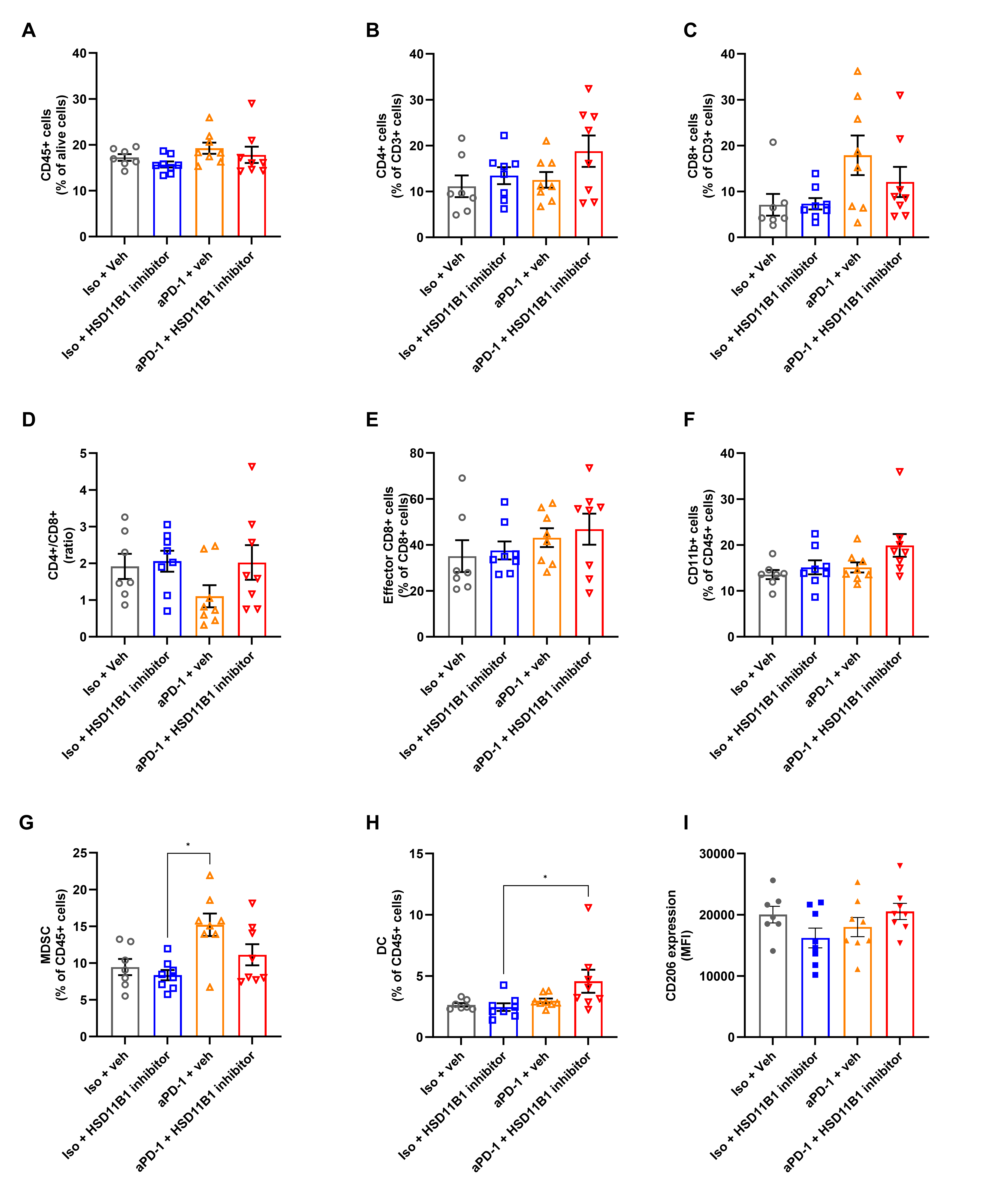

Supplement: Figure S6.tif [file KONI_A_2286820_SM8808.tif]
