# Supplementary material for: Activation of endogenous glucocorticoids by HSD11B1 inhibits the antitumor immune response in renal cancer
Source: Oncoimmunology. 2023 Nov 30;13(1):2286820. doi: 10.1080/2162402X.2023.2286820 (PMC10761155; doi:10.1080/2162402X.2023.2286820)
Supplement: Tables of supplementary material and method.docx [file KONI_A_2286820_SM8815.docx]

**Steroid hormone determination in kidney and plasma after cortisol-d_4_ injection**

| **Retention time (min)** | **Name** | **Prec Ion** **(*m/z*)** | **Prod Ion (*m/z*)** | **CE (V)** | **Polarity** |
| --- | --- | --- | --- | --- | --- |
| 6.21 | Aldosterone-d_7_ (internal standard) | 366.2 | 338.2 | 12 | NEG |
|  |  | 368.2 | 350.4 | 16 | POS |
| 6.60 | Cortisol-d_3_ | 366.2 | 121.1 | 24 | POS |
|  |  | 366.2 | 97.1 | 44 | POS |
| 6.61 | Cortisol-d_4_ | 367.2 | 121.1 | 24 | POS |
|  |  | 367.2 | 97.1 | 44 | POS |
| 6.65 | Cortisol | 363.2 | 121.0 | 36 | POS |
|  |  | 363.2 | 109.1 | 36 | POS |
| 6.65 | Cortisol-^13^C_3_ | 366.2 | 312.0 | 12 | POS |
|  |  | 366.2 | 124.1 | 24 | POS |
| 6.72 | Cortisone-d_3_ | 364.2 | 121.0 | 32 | POS |
|  |  | 364.2 | 97.0 | 40 | POS |
| 6.75 | Cortisone | 361.2 | 163.0 | 20 | POS |
|  |  | 361.2 | 121.0 | 28 | POS |
| 6.75 | Cortisone-^13^C_3_ | 364.2 | 166.0 | 24 | POS |
|  |  | 364.2 | 124.2 | 32 | POS |
| 7.58 | Corticosterone | 347.0 | 239.0 | 12 | POS |
|  |  | 347.0 | 112.0 | 36 | POS |
| 7.35 | 11-Dehydrocorticosterone | 345.0 | 121.1 | 28 | POS |
|  |  | 345.0 | 107.0 | 48 | POS |

**Cell media**

| **Medium** | **Product** | **Provider** | **Ref. number** |
| --- | --- | --- | --- |
| PBMC culture medium | RPMI | Gibco | 21875034 |
|  | 10 % FBS |  |  |
|  | 1 % MEM Non-Essential Amino Acids | Gibco | 11140035 |
|  | 10 mM Hepes | Gibco | 15630056 |
|  | 100 U/mL penicillin/streptomycin | Gibco | 15140122 |
|  | 2 mM L-glutamine | Gibco | 25030024 |
| PBMC freezing medium | PBMC culture medium |  |  |
|  | 40 % FBS |  |  |
|  | 10 % DMSO |  |  |
| FACS buffer | PBS |  |  |
|  | 0.5 % BSA |  |  |
|  | 2 mM EDTA |  |  |
| BMDC medium | RPMI | Gibco | 21875034 |
|  | 10 % FBS |  |  |
|  | 100 U/mL penicillin/streptomycin | Gibco | 15140122 |
|  | 2 mM L-glutamine | Gibco | 25030024 |
|  | 0.5 mM sodium pyruvate | Gibco | 11360039 |
|  | 50 μM 2-mercaptoethanol | Gibco | 31350010 |
| T cell medium | RPMI-Very low endotoxin | Bioswisstec AG | M3440 |
|  | 10 % FBS |  |  |
|  | 100 U/mL penicillin/streptomycin | Gibco | 15140122 |
|  | 2 mM L-glutamine | Gibco | 25030024 |
|  | 1 mM sodium pyruvate | Gibco | 11360039 |
|  | 1 % MEM Non-Essential Amino Acids | Gibco | 11140035 |
|  | 50 μM 2-mercaptoethanol | Gibco | 31350010 |
|  | 0.5 % BSA |  |  |
|  | 2 mM EDTA |  |  |

**FACS panels**

| **Panel** | **Antibody** | **Dilution in FACS buffer** | **Provider** | **Ref. number** |
| --- | --- | --- | --- | --- |
| mouse BMDC activation | anti-mouse CD16/32 | 1/100 | Biolegend | 101319 |
|  | anti-CD11c | 1/200 | Biolegend | 117331 |
|  | anti-CD80 | 1/200 | Biolegend | 104729 |
|  | anti-PD-L1 | 1/200 | Biolegend | 124331 |
|  | anti-MHC II | 1/200 | Miltenyi | 130-102-168 |
|  | anti-CD40 | 1/200 | eBioscience | 12-0401-82 |
|  | anti-CD11b | 1/200 | BD Bioscience | 550993 |
|  | anti-MHC I | 1/200 | eBioscience | 17-5958-80 |
|  | anti-CD86 | 1/200 | Biolegend | 105030 |
| mouse T cell purity | anti-mouse CD16/32 | 1/100 | Biolegend | 101319 |
|  | anti-CD3 | 1/200 | Biolegend | 100203 |
|  | anti-CD8a | 1/200 | Biolegend | 100708 |
|  | anti-CD4 | 1/200 | Biolegend | 100559 |
|  | anti-CD19 | 1/200 | Biolegend | 115512 |
| mouse tumor immunophenotyping: myeloid population markers | anti-mouse CD16/32 | 1/100 | Biolegend | 101319 |
|  | anti-CD45 | 1/200 | Biolegend | 103154 |
|  | anti-Ly6C | 1/200 | Biolegend | 128033 |
|  | anti-CD11b | 1/200 | Biolegend | 101233 |
|  | anti-IA/IE | 1/200 | Biolegend | 107641 |
|  | anti-CD206 | 1/200 | Biolegend | 141710 |
|  | anti-F4/80 | 1/200 | Biolegend | 123146 |
|  | anti-Ly6G | 1/200 | Biolegend | 127616 |
|  | anti-CD11c | 1/200 | Biolegend | 117318 |
| mouse tumor immunophenotyping: myeloid activation markers | anti-mouse CD16/32 | 1/100 | Biolegend | 101319 |
|  | anti-CD45 | 1/200 | Biolegend | 103154 |
|  | anti-CD11b | 1/200 | Biolegend | 101233 |
|  | anti-CD80 | 1/200 | Biolegend | 104729 |
|  | anti-IA/IE | 1/200 | Biolegend | 107641 |
|  | anti-CD86 | 1/200 | Biolegend | 105043 |
|  | anti-NKp46 | 1/200 | Biolegend | 137604 |
|  | anti-F4/80 | 1/200 | Biolegend | 123146 |
|  | anti-CD11c | 1/200 | Biolegend | 117318 |
|  | anti-CD83 | 1/200 | Biolegend | 121510 |
| mouse tumor immunophenotyping: lymphoid population markers | anti-mouse CD16/32 | 1/100 | Biolegend | 101319 |
|  | anti-CD45 | 1/200 | Biolegend | 103154 |
|  | anti-CD69 | 1/200 | Biolegend | 104532 |
|  | anti-CD44 | 1/200 | Biolegend | 103049 |
|  | anti-CD19 | 1/200 | Biolegend | 115543 |
|  | anti-CD3 | 1/200 | Biolegend | 100203 |
|  | anti-CD62L | 1/200 | Biolegend | 104408 |
|  | anti-CD8a | 1/200 | Biolegend | 100762 |
|  | anti-CD4 | 1/200 | Biolegend | 100422 |
|  | anti-CD25 | 1/200 | Biolegend | 102012 |

**Reagents**

| **Experiment** | **Product** | **Provider** | **Ref. number** |
| --- | --- | --- | --- |
| Immunohistochemistry | Polyclonal rabbit antibodies of HSD11B1 | Sigma | HPA 042186 |
| Multiplex immunohistochemistry | Thermoscientific Superfrost™ Gold Plus glass slides | Thermofischer, Massachusetts, USA |  |
| Human antigen recall assay | SepMate tubes | StemCell | 85450 |
|  | CMV | Astarte | 1004 |
|  | Tetanus toxoid | Astarte | 1002 |
|  | Tetanus toxoid | Calbiochem | 582231-25UG |
|  | Anti-PD-1, Humanized Antibody | BioVision | A1306 |
|  | Human IgG4, κ Isotype Control Antibody | BioVision | A1101 |
|  | Cortisone | Sigma | C2755 |
|  | ELISA MAX™ Standard Set Human IFN-γ | Biolegend | 430101 |
| Tumor-derived immune cell activation | MPLA | Avanti | 699800P-1MG |
| Genetic modification of Renca cells | Lipofectamine™ 2000 | Invitrogen | 11668019 |
| Differentiation and activation of BMDC | ELISA MAX™ Standard Set Mouse IL-6 | Biolegend | 431301 |
| Antigen-specific T cell-mediated tumor cytotoxicity assay | ovalbumine | Invivogen | Vac-pova-100 |
|  | CD8a+ T Cell Isolation Kit, mouse | Miltenyi | 130-104-075 |
|  | SIINFEKL | Invivogen | vac-sin |
| *In vivo* tumor model | R848 | Invivogen | tlrl-r848-5 |
|  | Surgical glue, VetBond |  | 1469SB |
| Steroid measurement in plasma | heparin tube | BD | 365966 |
|  | OasisPrime HLB 96-Well Plate | Waters, UK |  |
|  | ACQUITY UPLC HSS T3 Column | Waters, Switzerland |  |
| Tumor processing for immunophenotyping | Tumor Dissociation Kit, mouse | Miltenyi | 130-096-730 |
|  | Gentle MACS C tube | Miltenyi | 130-096-334 |
| FACS staining | Zombie Violet™ Fixable Viability Kit | Biolegend | 423113 |

**Instruments**

| **Experiment** | **Machine** | **Provider** |
| --- | --- | --- |
| Immunohistochemistry | Ventana System on Automates Benchmark Ultra | Roche |
| Multiplex immunohistochemistry | Pannoramic 250 Flash III scanner | 3D Histech, Budapest, Hungary |
| Preclinical MRI | nanoScan MRI 3T | Mediso,Medical Imaging Systems, Budapest, Hungary |
| Preclinical PET/CT imaging | Triumph preclinical PET/SPECT/CT | Trifoil Imaging, Chatsworth, USA |
| Steroid measurement in plasma | Vanquish UHPLC | Thermo Fisher Scientific, Reinach, Switzerland |
|  | Q Exactive Plus Orbitrap | Thermo Fisher Scientific, Reinach, Switzerland |
| Steroid measurement in kidney and plasma after cortisol-d_4_ injection | Agilent Infinity 1290 UHPLC system | Agilent Technologies, Santa Clara, US |
