# Supplementary material for: Activation of endogenous glucocorticoids by HSD11B1 inhibits the antitumor immune response in renal cancer
Source: Oncoimmunology. 2023 Nov 30;13(1):2286820. doi: 10.1080/2162402X.2023.2286820 (PMC10761155; doi:10.1080/2162402X.2023.2286820)
Supplement: Poinot et al Supplemental_text_2nd_rev.docx [file KONI_A_2286820_SM8812.docx]

Supplementary information to Poinot et al.

Supplementary figures

**Figure S1: The expression of HSD11B1 correlates with clinical outcome in patients with renal cancer.**

**A.** Steroidogenesis pathway. Steroids are represented as circle with their name in blue. Enzymes are represented in rectangles, in green for a correlation with good prognosis in RCC patients, in red for a correlation with poor prognosis in RCC patients. **B.** Kaplan-Meyer curves showing the probability of survival over time for ccRCC patients segregated based on the hierarchical clustering. The low *HSD11B1* expression group includes patients of Negative, *HSD11B2*, *CYP21A2* and *AKR1C4* clusters (Fig 1C). Statistical analysis: Log-rank (Mantel-Cox) test, *** p-value<10^-3^.

**Figure S2: Correlation between HSD11B1 expression and survival in multiple cancer types.**

Forest plot showing the hazard ratio (HR) for overall survival (OS) in cancer patients with high versus low expression of HSD11B1. Patients were segregated into high versus low HSD11B1 expression groups based on the median expression values as threshold by TIMER (Tumor IMmune Estimation Resource). The impact of the HSD11B1 expression level on OS was determined with Cox Proportional-Hazards model and the HR are represented with 95% confidence interval. HR is represented on a logarithmic scale, HR>1 indicates that high expression of HSD11B1 correlates with a shorter OS. Tested cancers were ranked by their p-value.

ACC: adrenocortical carcinoma, BLCA: bladder urothelial carcinoma, BRCA: breast invasive carcinoma, CESC: cervical and endocervical cancer, CHOL: cholangiocarcinoma, COAD: colon adenocarcinoma, DLBC: diffuse large B-cell lymphoma, ESCA: esophageal carcinoma, GBM: glioblastoma multiforme, HNSC: head and neck cancer, KICH: kidney chromophobe, KIRC: kidney renal clear cell carcinoma, KIRP: kidney renal papillary cell carcinoma, LGG: lower grade glioma, LIHC: liver hepatocellular carcinoma, LUAD: lung adenocarcinoma, LUSC: lung squamous cell carcinoma, MESO: mesothelioma, OV: ovarian serous cystadenocarcinoma, PAAD: pancreatic adenocarcinoma, PCPG: pheochromocytoma and paraganglioma, PRAD: prostate adenocarcinoma, READ: rectum adenocarcinoma, SARC: sarcoma, SKCM: skin cutaneous melanoma, STAD: stomach adenocarcinoma, TGCT: testicular germ cell tumors, THCA: thyroid carcinoma, THYM: thymoma, UCEC: uterine corpus endometrial carcinoma, UCS: uterine carcinosarcoma, UVM: uveal melanoma.

**Figure S3: Expression of HSD11B1 in tumor infiltrating cells.**

Immunohistochemistry slides of RCC patient tumor samples were analyzed for HSD11B1 expression by a pathologist. An HSD11B1 positive staining is represented by +, and negative staining by -. Macrophages and neutrophils were identified by their morphological characteristics as described in the supplementary material and methods. Follow-up period and grade of the disease are indicated for each patient. n.d.: not defined. n.a.: not applicable.

**Figure S4: Concentration response curve of ABT-384 in antigen recall assay.**

**A.** ABT-384 efficacy to reverse the effect of cortisone on IFN-γ levels secreted by PBMC from a healthy donor following antigen recall stimulation. Effect compared to the condition with cortisone, anti-PD-1 and with vehicle is represented as mean +/- SEM of 3 technical replicates in one experiment. EC50 of 0.31nM was calculated with nonlinear regression curve. Goodness of fit represented by R squared: 0.94. **B.** Concentration of IFN-γ levels secreted by PBMC following antigen recall stimulation. Data represented as mean +/- SEM of 3 technical replicates. Data are representative of 3 independent experiments on the same donor. Similar results were obtained with 2 other donors tested in one experiment.

**Figure S5: Gene expression of Hsd11b1 and Hsd11b2 in murine cell lines.**

Expression of *Hsd11b1* and *Hsd11b2* normalized with housekeeping gene (*HPRT*). Renca: renal cancer cell line, 4T1: breast cancer cell line, Hepa1-6: hepatoma cell line, MC38: colon cancer cell line, B16-F10: melanoma cell line.

**Figure S6: Effect of HSD11B1 inhibition on the tumor immune phenotype in anti-PD-1-treated subcutaneous Renca tumors.**

Immunophenotyping of the tumor by flow cytometry. Percentage of each subpopulation represented as % of live cells (**A**) or as % of CD45^+^ cells (**F**-**H**). Percentage of CD4^+^ and CD8^+^ represented as % of CD3^+^ cells (**B**-**C**) and as ratio of cell populations (**D**). CD44^-^CD62L^-^ CD8^+^ were defined as effector CD8^+^ cells and represented as % of CD8^+^ cells (**E**). CD206 expression (**I**) represented as mean fluorescence intensity (MFI) of CD206 on macrophages in the tumor. Data are shown as mean +/- SEM of 8 mice per group. * p-value≤0.05.

**Figure S7: HSD11B1 may improve the efficacy of anti PD-1 treatment in intrakidney tumor-bearing mice**

**A.** Growth of intra-renal Renca tumors of the experiment represented in figure 5 C-E. Treatment was initiated at day 7. Kaplan-Meyer curve showing the probability of survival over time. Survival was defined by the tumor metabolic activity measured by PET, with a PET signal over 2 considered as event of death. 10 mice per group. **B-E.** Relationship between corticosterone to 11-DHC ratio in the plasma and last tumor volume before death. 6 to 9 mice per group. Statistical analysis: Simple linear regression.

**Figure S8: Immunophenotyping of subcutaneous Renca tumors in mice treated with an HSD11B1 inhibitor and the innate immune-activating agent R848.**

Immunophenotyping results of the tumor by flow cytometry. Percentage of subpopulations represented as % of live cells (**A**), CD45^+^ cells (**F**-**H**), CD3^+^ cells (**B**, **C**), CD4^+^ cells (**D**) or CD8^+^ cells (**E**). Data are shown as mean +/- SEM of 7 to 8 mice per group. * p-value≤0.05, ** p-value<10^-2^.

**Figure S9: Gating strategies used for immunophenotyping of subcutaneous Renca tumors.**

Immunophenotyping results by flow cytometry of the tumor sample (number 3) of the experiment presented in Figure 6. **A.** Gating strategy of the “myeloid activation markers” antibody panel. NK cells were identified by NKp46 positive staining. Activation level of macrophages and DC were defined with CD80, CD86 and CD83 staining. **B.** Gating strategy of the “myeloid population markers” antibody panel. Population of MDSC, DC, and macrophages were identified as explained in the material and methods. **C.** Gating strategy of the “lymphoid population markers” antibody panel. B cells, CD4+ and CD8+ T cells, subpopulations of T cells and their level of activation were defined in this gating strategy.

**Supplementary materials and methods**

**Patient material**

The prognostic value of genes involved in steroidogenesis was tested in 533 clear cell renal cancer patients using RNA sequencing and clinical data from the TCGA. Genes involved in steroidogenesis were defined from the steroid hormone biosynthesis pathway from the KEGG pathway database. HSD11B1 staining was performed by immunohistochemistry on FFPE samples from tumor blocks of the primary tumors of ccRCC patients from the Geneva University Hospital biobank. 10 patients with poor prognosis and 10 patients with good prognosis were chosen to avoid a selection bias linked to disease stage. Survival data and stage of the disease are listed in Figure S3. For the activation assay of immune cells isolated from RCC patients, RCC tumors were collected by the surgeons at the Geneva University Hospital. The study methodology was reviewed by the Swiss Ethics Committees on Research Involving Humans and approved by the Commission Cantonale d’éthique de la recherche de Genève (2017-00364, to PNS).

**Immunohistochemistry**

Antigen retrieval was processed for 64 min with EDTA buffer (pH 8) and incubated for 32 min at 1:50 with HSD11B1 polyclonal rabbit antibodies. Primary antibodies were detected with anti-rabbit HRP complex and revealed by diaminobenzidine using automated routine procedures. Brown coloration corresponds to a positive staining.

Counterstained mounted slides were scanned, and HSD11B1 staining was quantitated with DEFINIENS software. Tumoral stained areas were considered as regions of interest (ROI) and were digitally marked. Then the algorithm counted the number of positively stained cells and the total number of cells in each selected ROI. The percentage of positive stained cells was calculated as follows: number of positive cells/total number of cells X 100.

For morphological analysis, macrophages were characterized as large round cells with a non-segmented nucleus shifted to the periphery and an apparent cytoplasm. Neutrophils were characterized as small cells with scant cytoplasm and a tri-lobulated nucleus.

**Multiplex immunohistochemistry**

FFPE tissue sections were cut at 4 μm and floated onto a 45 C water bath to be mounted on positively charged glass slides. After overnight drying, tissue sections were dewaxed by immersion in xylene, and rehydrated in ethanol of decreasing concentrations. After heat-mediated antigen retrieval of the slides in pH 6 citrate buffer (10 min), endogenous peroxidases, non-specific proteins, endogenous biotins, and avidins were blocked with corresponding blocking solutions from Dako™. Primary antibody was incubated on tissue sections, followed by a biotinylated secondary antibody and a streptavidin-HRP complex revealed by AEC chromogen. Slides were coated with a glass coverslip with an aqueous mounting solution and scanned into MRXS images. Glass coverslips were removed by immersion in hot water, and AEC staining washed in ethanol of increasing concentrations. Antibodies were stripped by boiling tissue sections in pH 6 citrate buffer for 10 to 20 min, and putative residual antibodies were blocked by Fab fragments directed against host of the previous primary antibody used. Then, multispectral immunohistochemistry was performed which consists of sequential cycles of staining with primary antibodies revealed by AEC chromogen, tissue section scanning, removal of AEC chromogen with ethanol, antibody stripping, and blocking with Fab fragments.

For the image processing, whole slide multiplexed images coming from the same tissue section were processed with an in-house code developed in Matlab R2022b (The MathWorks). Briefly, individual scans were aligned at a cellular level, according to the same reference image by using manual control points. Afterwards, hematoxylin and AEC immunohistochemical chromogenic staining of each image were unmixed and a single multiplexed image was exported.

**Human antigen recall assay**

PBMC were isolated from buffy coats of healthy donors (Centre de Transfusion Sanguine, Geneva, agreement n°758). Briefly, buffy coats were diluted by a factor 2 in PBS and centrifugated on a density gradient medium (Lymphoprep) at 1200 g for 30 min at room temperature using SepMate tubes. PBMC were collected above the density gradient and washed 3 times in PBS (400 g, 10 min, room temperature). After counting, PBMC were frozen at 1.5x10^7^ cells/mL for subsequent use (working on ice, freezing in Corning CoolCell Freezing container at -80 °C 24 h before storing in liquid N2) or tested for antigen response. Culture medium was filtered through 4 μm filters. The antigens were used at a final concentration of 1 or 0.1 μg/mL for CMV and 0.5 μg/mL for tetanus toxoid. Anti-PD-1 antibody and its isotype control were used at 1 or 0.1 μg/mL. Cortisone was used at a final concentration of 50 ng/mL. For the antigen recall assay, PBMC were thawed, carefully washed in culture medium (or used directly after isolation), counted, added at 2x10^5^ cells/well to a 96-well plate previously prepared with 2 X compounds, and incubated 6 days at 37 °C, 5 % CO_2_. Response to antigen was monitored via IFN-γ production measured by ELISA. In absence of cortisone, there was no effect of ABT-384 on the level of IFN-γ in stimulated (Fig S4 B) or non-stimulated conditions (data not shown).

**Activation assay of immune cells isolated from RCC patients.**

Tumors from RCC patients were collected from the surgeon, mechanically and enzymatically (collagenase I and IV) dissociated and immune cells were enriched with a density gradient centrifugation on Lymphoprep medium as for PBMC. After enrichment and washing, cells were seeded in a 96-well plate at 1.92x10^5^ cell/well in wells previously prepared with 2 X compounds. Culture medium was identical to the one used for PBMC, activation was done with a mix of antigens (0.1 μg/mL of CMV, 0.5 μg/mL of tetanus toxoid from Astarte, 0.5 μg/mL of tetanus toxoid from Calbiochem, 300 nM of MPLA), during 6 days of incubation at 37 °C 5 % CO_2_. Response to antigen was monitored via IFN-γ production measured by ELISA. One out of the four patients tested showed a response to antigen recall. The absence of T cell activation observed in the three other donors was attributed either to a low level of tumor immune cell infiltration or to the absence of reactivity against cytomegalovirus and tetanus toxoid antigens.

***Hsd11b1* and *Hsd11b2* gene expression in cell lines**

Renca, Hepa1-6, 4T1, MC38 and B16-F10 were frozen in cell pellet of 2x10^6^ cells for subsequent RNA extraction. After RNA extraction and reverse transcription using manufacturer protocols (Qiagen RNeasy Plus Mini Kit #74136, Applied Biosystems High Capacity cDNA Reverse Transcription Kit #4368814), cDNA was obtained and qPCR was performed (SYBR green method) with the following primers: *Hsd11b1* Fw 5’-CAGAAATGCTCCAGGGAAAGAA, *Hsd11b1* Rv 5’-GCAGTCAATACCACATGGGC, *Hsd11b2* Fw 5’-GGTTGTGACACTGGTTTTGGC, *Hsd11b2* Rv 5’-AGAACACGGCTGATGTCCTCT, *Hprt* Fw 5’-ATGAGCGCAAGTTGAATCTG, HPRT Rv 5’-CAGATGGCCACAGGACTAGA. Results are represented as mean of 2^(-ΔCt)^.

**Genetic modification of MHC H2-Kb Renca cell line**

The Renca cell line was genetically modified to express MHC H2-Kb Class I instead of MHC H2-Kd Class I normally express in BALB/c background. CRISPR/Cas9 was used to remove the H2-Kd gene and replace by the H2-Kb gene which was brought through a minicircle template as previously described.^2^ Modified Renca cells were termed Renca H2-Kb for further usage. Overexpression of *gfp* was performed on the Renca H2-Kb cells using lipofectamine transfection (manufacturer protocol) with plasmid containing *gfp* (plasmid #52961 from Addgene modified to replace *cas9* by *gfp*) and the cells renamed Renca H2-Kb GFP.

**Differentiation and activation of bone marrow-derived dendritic cells**

Bone marrow from 6 to 15-week-old C57BL/6 mice was flushed out of tibia and femur postmortem. After a 40 μm filtration, a red-blood-cell lysis was performed on the bone marrow cells. After washing and counting, the cells were incubated at 2.5x10^6^ cells/mL in 6-well plates with 20 ng/mL GM-CSF at 37 C 5 % CO_2_ for 2 days (BMDC medium in cell media table at the end of the materials and methods section). At day 2, half of the medium from each well was removed, centrifuged, and the cells resuspended in the same volume of fresh medium with 40 ng/mL GM-CSF and added back to their wells. At the end of day 3, the cells were collected with all the medium, centrifuged and resuspended in the double amount of fresh medium with 20 ng/mL GM-CSF and incubated for 3 additional days at 37 °C. At day 6 of the differentiation process, bone marrow-derived dendritic cells (BMDC) were collected, pooled, washed and distributed in 96-well plates at 1.5x10^5^ cells/well in presence of the following compounds for 24 or 48 h. R848 was used at a final concentration of 100 nM, the HSD11B1 inhibitor (BMS-823778 or ABT-384) at 1 μM, and 11-dehydrocorticosterone at 20 ng/mL. Cell surface proteins on BMDC were measured by flow cytometry with an antibody panel composed of antibodies against myeloid activation markers. IL-6 concentration in supernatant was measured by ELISA after 24 or 48 h of activation. DC were chosen as the most relevant and reliable model to study antigen cross-presentation *in vitro* compared to macrophages.

**Antigen-specific T cell-mediated cytotoxicity of tumor cells**

At day 6 of the BMDC differentiation, 2x10^4^ BMDC were seeded in 96-well plates in presence of the BMDC stimulation mix composed of R848 (100 nM) and ovalbumine (25 μg/mL) and treated with 11-DHC or vehicle and HSD11B1 inhibitor or vehicle. At day 7 of the BMDC differentiation, a spleen was collected from a 6 to 15-week-old TCR transgenic OT-I mouse (C57BL/6 background, Charles River), passed through a 40 μm cell strainer, and red blood cell lysis was performed. After washing and counting, CD8+ T cells were isolated through negative selection with a magnetic bead-based isolation kit following the manufacturer’s protocol and the purity was checked by flow cytometry. Isolated CD8+ T cells were added to the BMDC at 1x10^5^ cells/well in T cell medium after removing 50 % of the media. At day 9, H2-Kb-recombined Renca GFP+ cells were pulsed with 2 μg/mL of the ovalbumin antigenic peptide SIINFEKL (OVA_257–264_) for 1 h at 37 °C. 2.5x10^3^ cells were added to the co-culture wells. 3 hours after seeding, the confluency of Renca H2-Kb GFP was measured and used as baseline for further comparison. Renca H2-Kb GFP growth was then followed during 5 days with the Incucyte live-cell analysis system.

**Mice**

All animal experiments were authorized by Geneva cantonal authorities (Service de la consommation et des affaires vétérinaires) and followed the 3Rs principles to reduce, refine and replace animal experimentation. For subcutaneous tumor experiments, 6-week-old BALB/c mice were injected with 1x10^6^ Renca cells in 100 μL of PBS into the flank. After 5 to 7 days, tumors were palpable and measured with a caliper. Mice were assigned to treatment groups in order to obtain groups of comparable tumor area average, but were not moved from their initial cages to avoid stress and conflict. Area was measured 3 times per week.

For the orthotopic tumor experiment, 100 μL of 0.1 mg/kg buprenorphine was administered subcutaneously to 6-week-old BALB/c at least 20 min before the intervention. Mice were exposed to 2 % isoflurane for the induction of the anesthesia and 0.5 % to 1 % for the maintenance (in an individual mask on a heating pad). An incision of 0.5 to 0.7 cm was made in the skin of the mouse’s flank without opening the peritoneum. The experimenter localized the kidney, pulled and maintained it close to the skin incision, and slowly injected the cells. 10^5^ Renca cells in 10 μL were slowly injected into the kidney with a Hamilton syringe (needle of 30 G, 20 mm of length, bevel of 12 °) with the needle inserted at 30 ° at a depth of 4 mm (speed of injection around 1 μL/s, followed by a pause of 5 s before removing the needle). The incision was closed with surgical glue and mice checked twice a day for 3 days. 15 days after injection, tumor volumes of orthotopic renal cancer model were assessed with magnetic resonance imaging (MRI) and tumor activity was followed by [^18^F]FDG-PET/CT imaging once a week. To represent survival, tumor volume >200 mm^3^ was considered as event of death in figure 5A and PET signal >2 in figure S7A.

For both subcutaneous and intra-kidney tumor experiments, mice were treated as follow. Treatment of mice was initiated at day 7 after tumor cell injection. The HSD11B1 inhibitor ABT-384 or vehicle (0.5 % methylcellulose, 0.2 % Tween-80) were administered orally once a day in the morning at 10 mg/kg in 200 μL (with a PTFE feeding needle, 20 G, diameter 1.5 in., length 1.9 mm). Resiquimod (R848) or vehicle (PBS) were injected subcutaneously on two consecutive days repeated every 4 days, at 10 μg/mouse in 100 μL. Anti-PD-1 antibodies or isotype were injected intraperitoneally three times a week at 200 μg/mouse in 200 μL.

For MRI acquisitions, mice were exposed to 4 % isoflurane for the induction of the anesthesia and 0.5 to 2 % for the maintenance during the scan. 3T MRI acquisitions were performed with a mouse whole body transmit-receiver coil. T2-weighted fast spin echo FatSat images were acquired in the axial plane with acquisition parameters as follows: repetition time 7275 ms, echo time 104 ms, 10 excitations, field of view 40 × 40 mm^2^, acquisition matrix 128 × 128, spatial resolution 0.31 × 0.31 mm^2^, slice thickness 0.5 mm, no interslice gap, bandwidth 25,000 Hz, automatic fat saturation, scan durations 19 min 58 s.

For PET/CT acquisitions, mice were anesthetized with 4 % isoflurane and injected in the retro-orbital venous sinus with 4 to 5 megabecquerel (MBq) of [^18^F]FDG. Mice were then left awake for an uptake period of 60 min. Mice were anesthetized again with 4 % isoflurane and maintained with 2 % during the scans. Imaging chambers were heated to maintain a body temperature of 37 °C. PET and CT images were acquired on a preclinical PET/SPECT/CT scanner. CT images were obtained at 75 peak kilovoltage (kVp), 150 mA, and 1’024 projections were acquired during the 360 ° rotation with a field of view of 84.6 mm (1.4 magnification). PET scans were acquired for a total duration of 10 min. CT scans were reconstructed with the built-in Triumph XO software using a filtered back-projection algorithm with a matrix of 512 and a voxel size of 165 µm. PET scans were reconstructed with the built-in LabPET software using an OSEM3D (20 iterations) algorithm, and images were calibrated in Bq/mL by scanning a phantom cylinder. The Imalytics software version 3.0 (Gremse-IT GmbH, Aachen, Germany) was used to quantitatively analyze PET and MRI datasets. CT and PET scans were co-registered and PET series were converted to display standardized uptake values (SUVs) adjusted to the body weight of the animals. Maximum SUVs (maxSUVs) were quantified in the regions of interest corresponding to renal tumors and used in subsequent analyses. MRI images were used to quantify tumor volumes by tracing renal tumors.

**Steroid hormone determination in plasma**

To measure steroid hormone concentrations in the plasma, blood was terminally collected from mice through cardiac puncture immediately after CO_2_ euthanasia. Blood was collected in a heparin tube and kept on ice until centrifugation (2000 g, 10 min, 4 °C). The plasma corresponding to the upper phase was collected into clean tubes and snap frozen for mass spectrometric analysis of steroids^3^. Plasma samples were purified using solid phase extraction on an OasisPrime HLB 96-Well Plate. A Vanquish UHPLC (equipped with an ACQUITY UPLC HSS T3 Column, 100 Å, 1.8 µm, 1 mm x 100 mm column) was coupled to a Q Exactive Plus Orbitrap. Separation was achieved using gradient elution over 12 min using water and methanol both supplemented with 0.1 % formic acid (all Sigma-Aldrich, Buchs, Switzerland) as mobile phases. Data analysis was performed using TraceFinder 4.1 (Thermo Fisher Scientific, Reinach, Switzerland). Steroid hormone concentrations were calculated in nmol/L.

**Tumor collection**

Tumors were collected post mortem, weighed and divided for subsequent RNA analysis (snap freezing on dry ice) or flow cytometry analysis. To obtain cell suspensions for flow cytometry, tumors were digested in 1 mL of tumor dissociation enzyme mix in Gentle MACS C tubes with the program 37C_m_TDK_2 of the Gentle MACS Dissociator. After digestion, enzymes were inactivated with 10 mL of medium containing FBS, and the tumor suspension was filtered through a 40 μm cell strainer before washing in PBS and aliquoting in 96-well plates for subsequent staining. Each tumor suspension was separately stained with 3 different antibody panels: myeloid population markers, myeloid activation markers, or lymphoid population markers.

**Pharmacodynamics of ABT-384**

During 5 days, naive 6-week-old BALB/c males were treated with ABT-384 (synthetized by Spirochem) or vehicle (0.5 % methylcellulose, 0.2 % Tween-80) orally once daily in the morning at 10 mg/kg in 200 μL (with a PTFE feeding needle, 20 G, diameter 1.5 in., length 1.9 mm). The last day, mice were subcutaneously injected with 360 μg of cortisol-d4 (hydrocortisone-9,11,12,12-d4) in 200 μL (15 mg/kg, dilution at 1.8 mg/mL in PBS+DMSO (6.8 %)) 6 hours after ABT-384 treatment. Cortisol-d4 is converted into cortisone-d3 *in vivo* by HSD11B2 which is the substrate of HSD11B1. Cortisone-d3 is then converted into cortisol-d3 by HSD11B1. Conversion into cortisol-d3 was measured to assess HSD11B1 activity. Mice were euthanized 2.5 to 3.5 hours after cortisol-d4 injection and blood was collected post-mortem.

Measurement of cortisol-d3 after injection of cortisol-d4 allows an accurate measurement of HSD11B1 activity which is independent of the hypothalamic-pituitary-adrenal axis, excluding any bias linked to stress or potential compensation mechanisms by the adrenal gland in response to HSD11B1 inhibition. To avoid interference with the immune response and the tumor growth, this method was used only in naïve mice and measurement of the corticosterone/11-DHC ratio was used to evaluate the efficacy of the HSD11B1 inhibition in tumor experiments.

**Steroid hormone determination in kidney and plasma after cortisol-d_4_ injection**

Mouse kidneys were collected post mortem and frozen on dry ice. For liquid chromatography-mass spectrometry (LC-MS), 400 µL of aqueous 0.1 % formic acid were added to 150 mg of frozen tissue and homogenized with a tissue homogenizer (30 Hz, 2 min, 4 C). Then, 800 μL of cold acetonitrile were added to precipitate proteins. After vortexing (10 s) and centrifugation (10 min, 13000 rpm, 4 °C), 250 µL of supernatant were collected. 10 µL of internal standard solution (aldosterone-d7 at 1 µg/mL) were added and liquid-liquid extraction (LLE) was conducted by adding 0.5 mL of sodium phosphate buffer (1 M, pH 7), 2 mL of saturated NaCl, and 250 µL of K_2_CO_3_ (25 % w/v). After vortexing for 5 s, 6 mL of ethyl acetate were added and the tubes placed into a rotary mixer for (30 min, 35 rpm) for LLE extraction. After centrifugation (10 min, 3500 rpm, and 4 °C), the organic phase was transferred into fresh tubes and evaporated until dryness at reduced pressure. Samples were reconstituted with 100 µL of water:acetonitrile (50:50 v/v), centrifuged again (10 min, 13000 rpm, 4 °C) and supernatants were transferred to liquid chromatography (LC) vials for injection.

Plasma was collected as described above. Then, 10 µL of internal standard (aldosterone-d7 at 1 µg/mL) were added to 100 µL of plasma, and the samples were processed for LLE as for the kidney samples.

LC-MS measurements were performed on an Agilent Infinity 1290 UHPLC system consisting of a binary solvent delivery pump, a flexible cube module, a flow-through-needle autosampler, and a column oven. The LC was coupled to an Agilent G6490A triple quadrupole mass spectrometer through an Agilent Jet Stream ESI source. Data was acquired in dynamic multiple reaction monitoring (dMRM).

All separations were conducted on a Phenomenex core-shell column (Kinetex C18 100 Å, 2.1 x 150 mm, 1.7 µm, Phenomenex, Torrance, USA) equipped with the corresponding pre-column, and using water (A) and acetonitrile (B) as mobile phases, both containing 0.1 % formic acid and applying a gradient from 2 % to 100 % B in 14 min. The column temperature and flow rate were set at 30 °C and 300 µL·min^−1^, respectively.

Source conditions were: gas temperature 250 °C, gas flow 14 L·min^-1^, nebulizer pressure 20 psi, sheath gas heater 400 °C, sheath gas flow rate 11 L·min^-1^ and capillary voltage 3000 V.

Mass spectrometric conditions (MS/MS transitions, cone voltage, collision energy) were optimized using the Optimizer version B.08.00 software (Agilent Technologies, Santa Clara, US) by flow injection analysis of individual steroid standard (1 µg⋅mL^-1^) using a mixture of water:acetonitrile (50:50 v/v) containing 0.1 % formic acid at a flow rate of 100 µL·min^−1^. The obtained precursor and product ions, the collision energies (CE) and retention times monitored for each compound are reported in the table below. For all transitions, precursor and product ion selection was performed with a resolution of 0.7 Da. Two transitions (quantifier and qualifier) of the main isotopic form for analytes and internal standard were selected for quantification and confirmation. Data acquisition and instrument control were performed using MassHunter version B.08.00 (Agilent Technologies, Santa Clara, US).

The y-axis of Figure 4A and 5B represents the intensity of the mass spectrometry signal of cortisol-d3 corrected by the one from the internal standard (aldosterone-d7). Raw values for steroid quantification were normalized by the weight of each kidney tissue sample.

**Flow cytometry**

Cells were stained with a Zombie viability marker in PBS during 20 min at 4 °C in the dark. After PBS wash (400 g, 5 min, 4 °C), the cells were resuspended in the antibody panels and incubated 15 min at 4 °C. Samples were read with a Novocyte3000 (violet, blue and red lasers, 13 colors) and analysis done with NovoExpress Software.

Gating strategies for the 3 antibody panels are shown in Figure S9. MDSCs were defined as CD45+ Ly6C+ and removed from the following analysis as presented in the gating strategy of the “myeloid population markers” panel (Figure S9B). Macrophages were defined as CD45+ CD11b+ CD11c- F4/80+ as presented in the gating strategy of the “myeloid population markers” panel (Figure S9B). DC were defined as CD45+ CD11b+ CD11c+ as presented in the gating strategy of the “myeloid population markers” panel (Figure S9B).

**Statistical analyses**

All statistical analyses were performed using GraphPad Prism software except the hierarchical clusters and heat maps which were generated by using TIBCO Spotfire.

For the TCGA data analyses, patients were dichotomized into two groups based on the high versus low expression of each individual genes (median as threshold). The impact of the expression level on overall survival (OS) was assessed using the Cox proportional-hazards model in GraphPad Prism. All the analyzed genes were ranked according to their p-value and plotted with their hazard ratio (HR) generated by the Cox regression model. HR were used to estimate the probability of an event (death of the patient) occurring in the high and low group of patients. HR are represented with 95 % confidence interval. A HR > 1 favors patients with low gene expression. In hierarchical clustering analysis, patients were ranked according to the level of expression of genes involved in glucocorticoid metabolism (*AKR1C4*, *CYP21A2*, *HSD11B1*, *HSD11B2*). The Ward’s clustering method was used, with half-square Euclidean for the distance measure, mean value for the ordering of gene expression, and Z score calculation for the normalization and correlation of OS was shown using Kaplan-Meyer plot based on the identified clusters. A Gehan-Breslow-Wilcoxon test was used to assess the difference of survival between the groups. Immune infiltrate analysis was performed on the five clusters of patients already identified by hierarchical clustering analysis. For this, the mRNA expression of immune checkpoints (*PDCD-1*, *LAG-3*, and *CTLA-4*) was evaluated in each patient as well as a Th2 gene signature.^1^ The expression levels for immune checkpoints and the Th2 gene signature in patients with high vs low *HSD11B1* expression were compared with a multiple unpaired t test, two-stage step-up, desired FDR = 1 %.

For all the comparisons of quantitative variables in conditions involving more than one categorical variable, data were analyzed with ordinary two-way ANOVA, alpha 0.05, Tukey’s multiple comparison tests (IFN-γ concentrations in Fig 3 A and B, tumor surfaces in Fig 4 B and Fig 6 A, expression of CD86 and MHCII and percentage of growth in Fig 7 C, D and E).

For parameters not following normal distribution, a Wilcoxon test with two-tailed p-value was performed (corticosterone/11-DHC ratio in plasma and kidney in Fig 5 A). When multiple comparisons were needed, a Kruskal-Wallis test were performed with Dunn’s multiple comparison tests (immunophenotyping results of the tumor in Fig 4 C and D, and in Fig 6 B-D, corticosterone/11-DHC ratio in plasma in Fig 5 D).

For parameters following normal distribution, an unpaired t test with two-tailed p-value was performed (cortisol-d_3_ levels in plasma (Fig4 A) and in kidneys (Fig 5 B)).

**Cell media, FACS panels, Reagents, Instruments**

See tables in supplementary material

**References:**

[1] Bindea G, Mlecnik B, Tosolini M, Kirilovsky A, Waldner M, Obenauf AC, Angell H, Fredriksen T, Lafontaine L, Berger A, et al. Spatiotemporal Dynamics of Intratumoral Immune Cells Reveal the Immune Landscape in Human Cancer. *Immunity*. 2013;39(4):782-795. doi:10.1016/j.immuni.2013.10.003

[2] Kelton W, Waindok AC, Pesch T, Pogson M, Ford K, Parola C, Reddy ST. Reprogramming MHC specificity by CRISPR-Cas9-assisted cassette exchange. *Sci Rep*. 2017;7(1):45775. doi:10.1038/srep45775

[3] Andrieu T, du Toit T, Vogt B, Mueller MD, Groessl M. Parallel targeted and non-targeted quantitative analysis of steroids in human serum and peritoneal fluid by liquid chromatography high-resolution mass spectrometry. *Anal Bioanal Chem*. 2022;414(25):7461-7472. doi:10.1007/s00216-022-03881-3
