# Supplementary material for: Activation of endogenous glucocorticoids by HSD11B1 inhibits the antitumor immune response in renal cancer
Source: Oncoimmunology. 2023 Nov 30;13(1):2286820. doi: 10.1080/2162402X.2023.2286820 (PMC10761155; doi:10.1080/2162402X.2023.2286820)
Supplement: Figure S3.docx [file KONI_A_2286820_SM8809.docx]

| HSD11B1 staining | |  |  |  |  |  |
| --- | --- | --- | --- | --- | --- | --- |
| ID patients | Macrophages | Neutrophils | HSD11B1 staining location | | Follow-up period (days) | Grade |
|  |  |  | Core | Margin |  |  |
| RCC 6 | + | n.a. | + | - | 7019 | 1 |
| RCC 23 | - | - | n.d. | n.d. | 7215 | 1 |
| RCC 24 | - | - | n.d. | n.d. | 6511 | 1 |
| RCC 30 | + | - | + | - | 8865 | 1 |
| RCC 32 | + | + | + | - | 730 | 3 |
| RCC 44 | - | - | + | - | 5615 | 1 |
| RCC 45 | + | + | + | - | 921 | 3 |
| RCC 58 | + | + | + | - | 372 | 4 |
| RCC 59 | + | + | n.d. | n.d. | 50 | 3 |
| RCC 66 | - | - | n.d. | n.d. | 5175 | 1 |
| RCC 71 | - | - | + | - | 5396 | 1 |
| RCC 78 | - | - | n.d. | n.d. | 192 | 3 |
| RCC 84 | - | - | n.d. | n.d. | 493 | 3 |
| RCC 91 | - | - | n.d. | n.d. | 4962 | 1 |
| RCC 97 | - | - | n.d. | n.d. | 5828 | 1 |
| RCC 107 | - | - | n.d. | n.d. | 162 | 3 |
| RCC 117 | - | - | + | n.a. | 656 | 3 |
| RCC 119 | - | - | n.d. | n.d. | 2567 | 3 |
| RCC 137 | + | + | + | + | 5302 | 1 |
| RCC 139 | - | - | n.d. | n.d. | 3814 | 3 |
